# Supplementary material for: Refinement of pore size at sub-angstrom precision in robust metal–organic frameworks for separation of xylenes
Source: Nat Commun. 2020 Aug 27;11:4280. doi: 10.1038/s41467-020-17640-4 (PMC7453017; doi:10.1038/s41467-020-17640-4)
Supplement: Supplementary file 1 — Supplementary Information [file 41467_2020_17640_MOESM1_ESM.pdf]

Supplementary Information

Refinement of pore size at sub-angstrom precision in robust metal-organic frameworks for separation of xylenes

Li et al.

Supplementary Methods

Material synthesis.

Preparation of MFM-300(V): H_4L (biphenyl-3,3′,5,5′-tetracarboxylic acid 0.14 g, 4.2 mmol) and VCl_3 (0.4 g, 2.5 mmol) were mixed and dispersed in 20 mL deionized water in a 45 mL autoclave. The slurry was acidified by adding 2 mL HCl (1% wt), and the autoclave was sealed and heated at 210 ºC for 3 days. The resultant green precipitate was collected, exchanged with acetone and dried.

Preparation of MFM-300(In): H_4L (biphenyl-3,3′,5,5′-tetracarboxylic acid 1.12 g, 3.4 mmol) and $In(NO_3)_3{\cdot}9H_2O$ (3.13 g, 7.6 mmol) were mixed and dispersed in a DMF/MeCN mixture (100 mL,3:1 v/v) in a 250 mL glass pressure reactor. The white slurry was acidified with conc. nitric acid (65% 2.5 mL), and the vessel sealed and heated at 90 °C for 48 h. The resultant flaky white precipitate was collected, exchanged with acetone and dried in air.

***Preparation of MFM-300(Al)*:** H_4L (H_4L = biphenyl-3,3′,5,5′-tetracarboxylic acid, 2.0 g, 6.1 mmol), $AlCl_3{\cdot}6H_2O$ (4.0 g, 16.6 mmol) were mixed and dispersed in DMF (125 mL), and HCl (10% wt, 25 mL) was then added to the resultant white slurry. The slurry was transferred into a 300 mL autoclave, which was sealed and heated to 145ºC for 2 days. After cooling over 12 h to room temperature, the resultant white microcrystalline product was separated by filtration, exchanged with acetone and dried in air.

***Preparation of MFM-300(Fe)*:** H_4L (H_4L = biphenyl-3,3′,5,5′-tetracarboxylic acid, 2.44 g, 7.4 mmol), $FeCl_3{\cdot}6H_2O$ (8.0 g, 29.6 mmol) were mixed and dispersed in DMF (200 mL) solution, and conc. HCl (10 mL) was then added to the resultant yellow slurry. The slurry was transferred into a 500 mL round-bottom flask, which was heated to 120 ºC for 3 days. The resultant yellow powder was collected, exchanged with acetone and dried in air.

Powder X-ray diffraction: Powder X-ray diffraction (PXRD) data were collected over the 2θ range 3-50º on a Bruker Advance D8 diffractometer using Cu-$K_{\alpha 1}$ radiation (λ = 1.54056 Å, 40 kV/30mA).

N_2 adsorption isotherms: The acetone-exchanged samples of MFM-300 were degassed at 150 °C and 10^{-10} bar for 12 h to yield the fully desolvated samples, which were then loaded for characterization of porosity. N_2 adsorption isotherms were recorded at 77 K using liquid nitrogen.

Synchrotron terahertz spectroscopy: The terahertz spectroscopy of xylene-loaded MFM-300(In) was measured in a Bruker Vertex 80V spectrometer with a liquid helium cooled silicon bolometer detector at the MIRIAM Beamline B22 of Diamond Light Source. The beam splitter in the FTIR was a 6 µm thick multilayer mylar with a nominal transmission range from 680-30 cm^{-1}. Typically, 512 scans were co-averaged per spectrum with an FTIR scanner velocity of 40 kHz at the reference laser wavenumber (15799 cm^{-1}). The spectral resolution was 2 cm^{-1}.

Molecular dynamics modelling: Molecular dynamics (MD) simulations were performed using CP2K (http://www.cp2k.org)[1], based on the mixed Gaussian and plane-wave scheme1[2] and the Quickstep module[3]. The calculation used molecularly optimized Double-Zeta-Valence plus Polarization (DZVP) basis set[4], Goedecker-Teter-Hutter pseudopotentials[5], and the Perdew-Burke-Ernzerhof (PBE) exchange correlation functional[6]. The plane-wave energy cutoff was 400 Ry. The DFT-D3 level correction for dispersion interactions, as implemented by Grimme et al[7,] was applied, with a cutoff distance of 15 Å. To simulate the transport of molecules inside the pores, a 1×1×2 supercell of the experimentally determined unit cell was first created. The supercell contains 432 atoms, with 8 xylene molecules in the pores. The metal centres in the MOF were fixed during the MD simulation to ensure that the framework does not drift on incorporation of the guest molecules; all other atoms in the ligands and xylene molecules were allowed to move freely. The temperature of the molecules were set to 300 K controlled by the Nose thermostat. A small constant force of 0.001 eV $Å^{-1}$ along z-direction (the direction along the pores) was applied to the molecules (and only these molecules) to simulate transport of the molecules under a potential gradient.

3D X-ray imaging: The packed MFM-300(In) stainless steel column was scanned directly using a Zeiss Xradia 520 X-ray Microscope system housed in a customize bay at the Henry Moseley X-Ray Imaging Facility (HMXIF). The distance between the specimen and source was 72.706 mm and the distance between the specimen and the detector was 8.005 mm. The column was rotated over a 360

degrees over 2 days while collecting 1601 projections per scan with a voxel size of 0.6128 μm. The accelerating voltage was 100 kV and 90 μA with an exposure of 100 ms for each projection at an optical magnification of 20x. After image acquisition, data sets were reconstructed using the Scout-and-Scan Control System Reconstructor (11.1.5707.17179) and data were loaded into Avizo standard 7.0 (Visualization Sciences Group, Bordeaux (VSG), France) for examination of the virtual slices and 3D volume renderings.

Batch adsorption experiments: Liquid phase batch adsorption experiments were carried out at room temperature in 1.8 mL glass vials containing 0.005 g of MFM-300 and 1 mL of a single compound solution in pentane. The equilibrium time for each xylene isomer on these materials was 2 h. As an aliphatic solvent is unlikely to interact strongly with the host material[9-11], it can be assumed that the choice of aliphatic solvent will not influence significantly the adsorption capacity or separation factor for the various alkylaromatics. Uptakes were directly calculated after 2h from the GC output data. The amount adsorbed q was calculated using **eq. 1**.

$$q = \frac{C_0 - C_t}{m} \qquad \textbf{(eq. 1)}$$

where C_0 and C_t represent the solution concentration before and after adsorption, and m is the mass of adsorbent.

Vapor adsorption experiments: Single component isotherms for xylene adsorption in MFM-300 were recorded at 318.15 K using a temperature controlled water bath on a Hiden IGA under ultrahigh vacuum. The solvents were degassed in the vapour-loading chamber of the IGA. In each case around 80 mg of MFM-300(M) (M = Al, V, In) was loaded into the IGA and activated under ultrahigh vacuum at 423 K for 24 h. Between runs the samples were reactivated at 423 K for 24 h.

Supplementary Table 1. BET surface areas of MFM-300s before and after use for xylene separation.

Materials	Surface area (m^2 g^{-1})	Pore volume (cm^3 g^{-1})[a]	Pore size (Å)[a]	Surface area (m^2 g^{-1})[b]
MFM-300(In)	903	0.31	7.4	880
MFM-300(V)	1091	0.35	6.9	1033
MFM-300(Fe)	924	0.25	6.8	863
MFM-300(Al)	1139	0.39	6.5	1108

[a] Calculated based on H-K model.

[b] Used MFM-300 samples post xylene separations.

Supplementary Table 2 Summary of bed void times and retention times in all MOF columns in this work.

Column	t_m(min)	t_{rpX} (min)	t_{roX} (min)	t_{rmX} (min)
MFM-300(In)	5	15	21	51
MFM-300(V)	4	5	9	19
MFM-300(Fe)	4	5	7	22
MFM-300(Al)	3	3	4	4
MFM-300(In-V)	10	22	39	90

Supplementary Table 3. Selectivities for xylene isomers in MFM-300[a].

MOFs	xylene isomers	Selectivity[b]	Selectivity[c]	Selectivity[d]
MFM-300(In)	*m*-xylene/*p*-xylene	3.5	3.0	3.8
	m-xylene/*o*-xylene	2.3	2.1	2.7
	o-xylene/*p*-xylene	1.3	1.5	1.4
MFM-300(V)	*m*-xylene/*p*-xylene	3.7	2.3	1.6
	m-xylene/*o*-xylene	1.1	1.5	1.2
	o-xylene/*p*-xylene	3.0	1.5	1.3
MFM-300(Fe)	*m*-xylene/*p*-xylene	3.9	3.9	3.0
	m-xylene/*o*-xylene	1.6	1.4	1.5
	o-xylene/*p*-xylene	2.5	2.8	2.0
MFM-300(In-V)	*m*-xylene/*p*-xylene	-	2.7	-
	m-xylene/*o*-xylene	-	1.8	-
	o-xylene/*p*-xylene	-	1.5	-

[a] all selectivities were calculated according to experiments carried out at 293K.

[b] selectivities calculated from binary breakthrough curves.

[c] selectivities calculated from15mM ternary breakthrough curves.

[d] selectivities calculated from 50mM ternary breakthrough curves.

Supplementary Table 4. Summary of crystal data from PXRD refinements of xylene-loaded MFM-300(V).

	MFM-300(V)·p-xylene	MFM-300(V)·o-xylene	MFM-300(V)·m-xylene
Formula	$C_{16}H_8V_2O_{10}[C_8H_{10}].1.88H_2O$	$C_{16}H_8V_2O_{10}[C_8H_{10}].2.36\ H_2O$	$C_{16}H_8V_2O_{10}[C_8H_{10}].2.45\ H_2O$
Formula weight (g mol^{-1})	602.12	610.76	612.38
Temp/K	298	298	298
Radiation type	Synchrotron	Synchrotron	Synchrotron
Diffractometer	Beamline I11	Beamline I11	Beamline I11
Data collection mode	Transmission	Transmission	Transmission
Wavelength(Å)	0.825633	0.825868	0.825633
Crystal system	Tetragonal	Tetragonal	Tetragonal
Space group	$I4_122$	$I4_122$	$I4_122$
a / Å	15.057(3)	15.05114(10)	15.05714(12)
b / Å	15.057(3)	15.05114(10)	15.05714(12)
c / Å	11.972(2)	11.97156(12)	11.97880(11)
V/ Å^3	2715.23(6)	2712.00(4)	2715.80(5)
D_c /g cm^{-3}	1.58	1.48	1.49
R_{exp} / %	4.33	4.89	4.48
R_{wp} / %	9.17	7.68	8.19
R_p / %	7.06	6.01	6.31
GoF	2.12	1.57	1.83
R_{Bragg}	2.07	3.80	4.40
CCDC deposition number	1828526	1828525	1828524

Supplementary Table 5. Summary of crystal data from PXRD refinements of xylene-loaded MFM-300(In).

	MFM-300(In)·p-xylene	MFM-300(In)·o-xylene	MFM-300(In)·m-xylene
Formula	$C_{16}H_8In_2O_{10}[C_8H_{10}].2H_2O$	$C_{16}H_8In_2O_{10}[C_8H_{10}].2H_2O$	$C_{16}H_8In_2O_{10}[C_8H_{10}].4H_2O$
Formula weight (g mol^{-1})	732.03	732.03	768.03
Temp/K	298	298	298
Radiation type	Synchrotron	Synchrotron	Synchrotron
Diffractometer	Beamline I11	Beamline I11	Beamline I11
Data collection mode	Transmission	Transmission	Transmission
Wavelength(Å)	0.825868	0.825868	0.825868
Crystal system	Orthorhombic	Tetragonal	Tetragonal
Space group	$I2_12_12_1$	$I4_122$	$I4_122$
a / Å	15.2829(2)	15.497755(12)	15.475320(15)
b / Å	15.2829(2)	15.497755(12)	15.475320(15)
c / Å	12.32365(15)	12.328602(8)	12.331478(9)
V/ Å^3	2964.47(7)	2961.089(5)	2953.210(6)
D_c /g cm^{-3}	1.63	1.63	1.71
R_{exp} / %	3.32	3.39	3.67
R_{wp} / %	15.21	9.68	9.30
R_p / %	11.16	7.53	6.82
GoF	4.58	2.85	2.53
R_{Bragg}	5.94	6.15	3.76
CCDC deposition number	1828523	1828522	1828521

Supplementary Table 6. Summary of crystal data from PXRD refinements of xylene-loaded MFM-300(Fe).

	MFM-300(Fe)·p-xylene	MFM-300(Fe)·o-xylene	MFM-300(Fe)·m-xylene
Formula	$C_{16}H_8Fe_2O_{10}[C_8H_{10}].2H_2O$	$C_{16}H_8Fe_2O_{10}[C_8H_{10}].1.38H_2O$	$C_{16}H_8Fe_2O_{10}[C_8H_{10}].2.78H_2O$
Formula weight (g mol^{-1})	614.08	602.92	628.12
Temp/K	298	298	298
Radiation type	Synchrotron	Synchrotron	Synchrotron
Diffractometer	Beamline I11	Beamline I11	Beamline I11
Data collection mode	Transmission	Transmission	Transmission
Wavelength(Å)	0.826013	0.826013	0.826013
Crystal system	Tetragonal	Tetragonal	Tetragonal
Space group	$I4_122$	$P4_122$	$I4_122$
a / Å	15.11950(14)	15.12259(3)	15.12059(3)
b / Å	15.11950(14)	15.12259(3)	15.12059(3)
c / Å	12.09146(9)	12.09135(2)	12.09837(2)
V/ Å^3	2764.10(5)	2765.205(12)	2766.077(13)
D_c /g cm^{-3}	1.47	1.44	1.49
R_{exp} / %	3.92	3.79	4.21
R_{wp} / %	10.27	8.51	6.72
R_p / %	7.44	6.39	5.11
GoF	2.62	2.24	1.60
R_{Bragg}	3.60	5.31	2.04
CCDC deposition number	1828520	1828519	1828518

Supplementary Table 7. Summary of structural data from PXRD refinements of xylene-loaded MFM-300(Al).

	MFM-300(Al)·p-xylene	MFM-300(Al)·o-xylene	MFM-300(Al)·m-xylene
Formula	$C_{16}H_8Al_2O_{10}[C_8H_{10}].4H_2O$	$C_{16}H_8Al_2O_{10}[C_8H_{10}].2H_2O$	$C_{16}H_8Al_2O_{10}[C_8H_{10}].3.7H_2O$
Formula weight (g mol^{-1})	592.36	556.36	586.96
Temp/K	298	298	298
Radiation type	Synchrotron	Synchrotron	Synchrotron
Diffractometer	Beamline I11	Beamline I11	Beamline I11
Data collection mode	Transmission	Transmission	Transmission
Wavelength(Å)	0.825706	0.826162	0.825706
Crystal system	Tetragonal	Tetragonal	Tetragonal
Space group	$I4_122$	$I4_122$	$I4_122$
a / Å	14.79676(3)	14.82712(6)	14.79571(3)
b / Å	14.79676(3)	14.82712(6)	14.79571(3)
c / Å	11.76485(3)	11.80007(6)	11.76322(3)
V/ Å^3	2575.843(13)	2594.17(2)	2575.124(13)
D_c /g cm^{-3}	1.50	1.41	1.50
R_{exp} / %	3.64	0.88	4.66
R_{wp} / %	11.25	8.09	12.17
R_p / %	7.67	5.60	8.40
GoF	3.09	9.15	2.61
R_{Bragg}	9.46	4.39	8.66
CCDC deposition number	1828517	1828516	1828527

Supplementary Table 8. Summary of distances for host-guest interactions between xylene and MFM-300.

Materials	Guest molecules	$\pi\cdots\pi$ (Å)	$\pi\cdots O$ (Å)
MFM-300(In)	*p*-xylene	5.47(5)	4.57(1)
	o-xylene	4.25(9)	4.57(1)
	m-xylene	4.25(1)	3.77(2)
MFM-300(V)	*p*-xylene	3.98(2)	3.98(4)
	o-xylene	3.75(8)	4.28(2)
	m-xylene	4.01(8)	3.78(1)
MFM-300(Fe)	*p*-xylene	4.12(2)	-
	o-xylene	3.79(8)	-
	m-xylene	3.73(9)	-
MFM-300(Al)	*p*-xylene	3.74(2)	-
	o-xylene	3.53(5)	-
	m-xylene	3.53(8)	-

Supplementary Table 9. Conditions for the selectivity data calculated for various MOFs.

Materials	Temperature (K)	Flow rate (mL min^{-1})	Concentration (M)	Selectivies calculated from	Ref.
MFM-300(In)	293	0.5	0.015	ternary breakthrough curves	This work
MFM-300(V)	293	0.5	0.015	ternary breakthrough curves	This work
MFM-300(Al)	293	0.5	0.015	ternary breakthrough curves	This work
MFM-300(In-V)	293	0.5	0.015	ternary breakthrough curves	This work
MIL-125(Ti)-NH_2	298	-	0.024	binary breakthrough curves	9
UiO-66(Zr)	313	0.25	0.08	ternary breakthrough curves	10
MIL-53(Fe)	323	1.0	0.005	binary breakthrough curves	11
MIL-47(V)	298	-	0.028	binary liquid-phase batch adsorption curves	12
MIL-53(Al)-ht	298	-	0.028	binary liquid-phase batch adsorption curves	12
[$Cu_3(BTC)_2$]	298	-	0.028	binary liquid-phase batch adsorption curves	12
$Co_2(dobdc)_2$	306	-	0.01-1.7	multicomponent liquid-phase isotherms	13

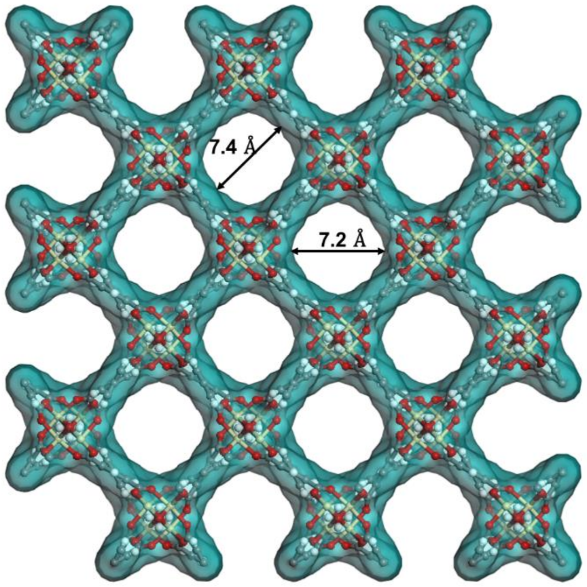

a

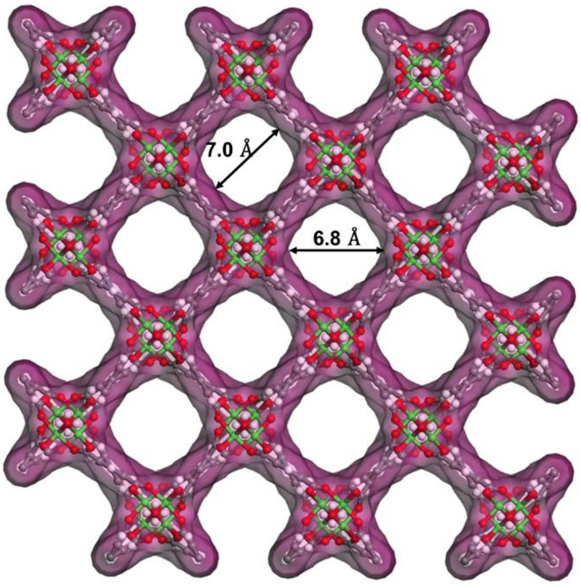

b

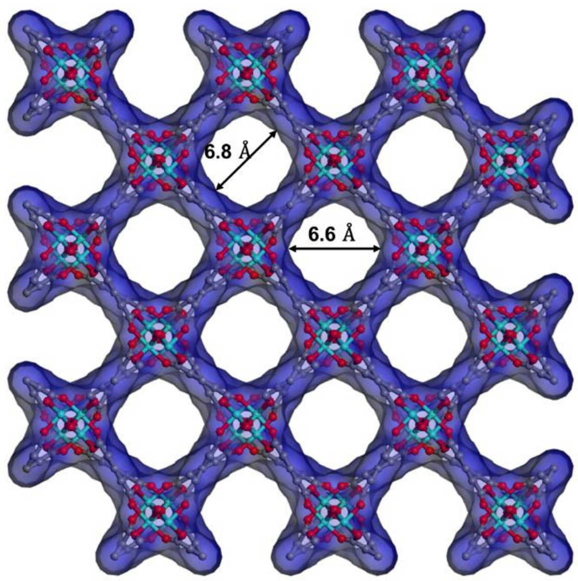

c

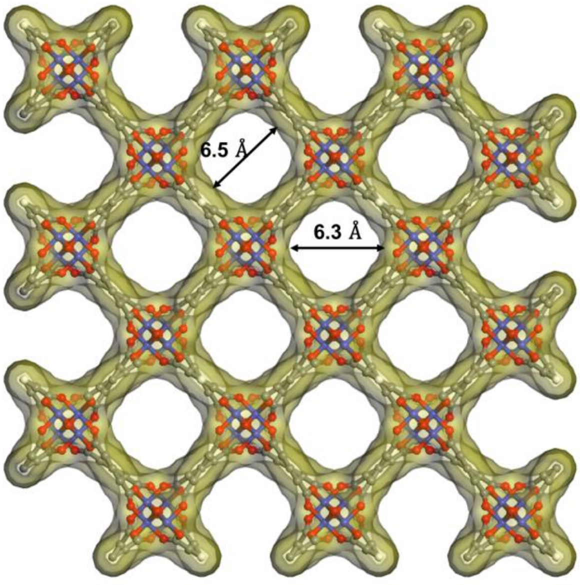

d

Supplementary Fig. 1. Representation of the crystal structure of **a,** MFM-300(In), **b,** MFM-300(V), **c,** MFM-300(Fe), **d,** MFM-300(Al) showing microporous square-shaped channels in a 3×3×4 super cell along the c-axis. The van der Waals surface is shown in green, magenta, purple and yellow, respectively.

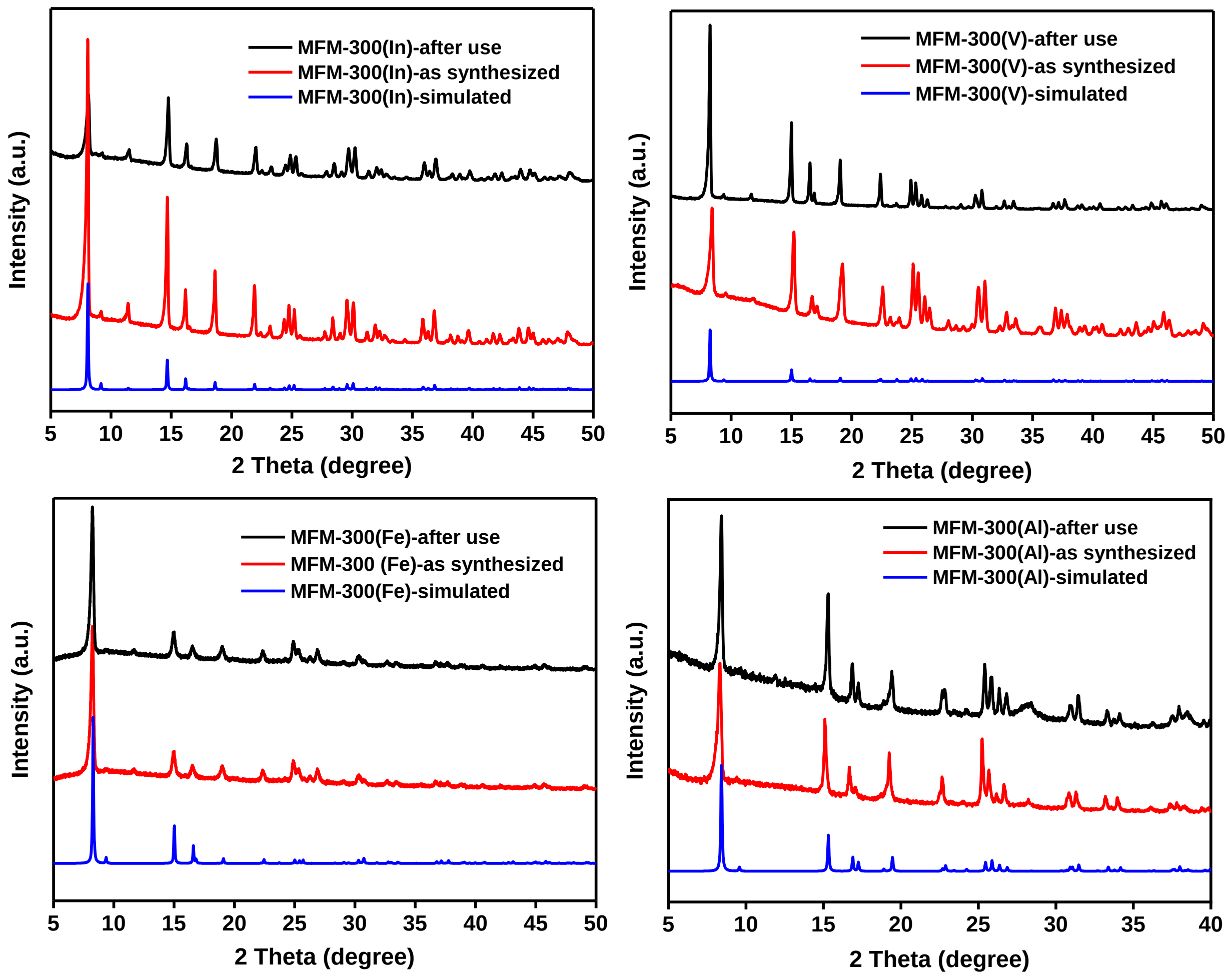

Supplementary Fig 2. PXRD patterns for MFM-300 before and after the xylene separation.

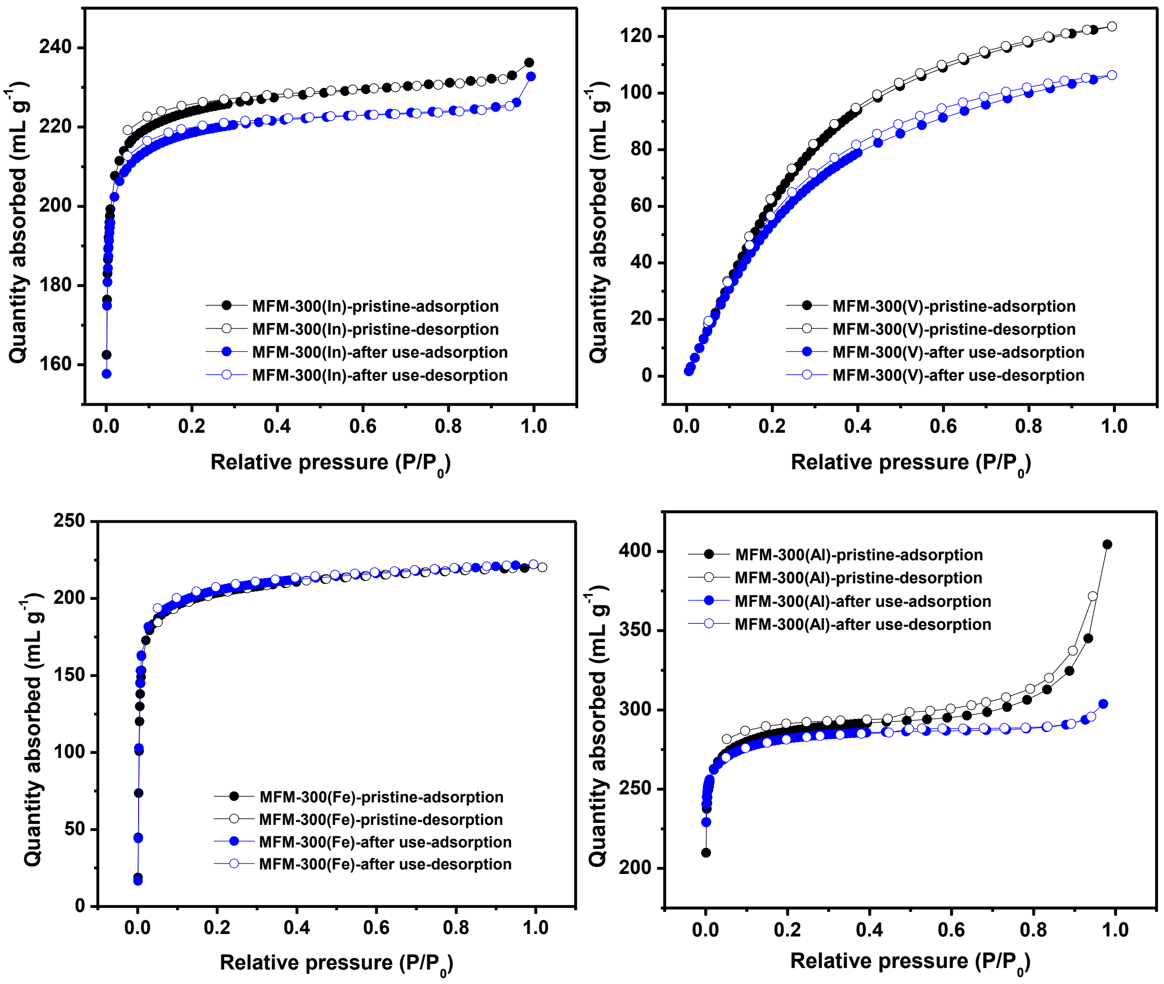

Supplementary Fig. 3. N_2 (77K) adsorption isotherms for MFM-300(In, Al) and CO_2 (273 K and 195 K) adsorption isotherms for MFM-300(V and Fe), respectively. CO_2 adsoption was used in cases where diffusion of N_2 at 77 is too slow to achieve adsorption equilibrium in a reasonable timescale.

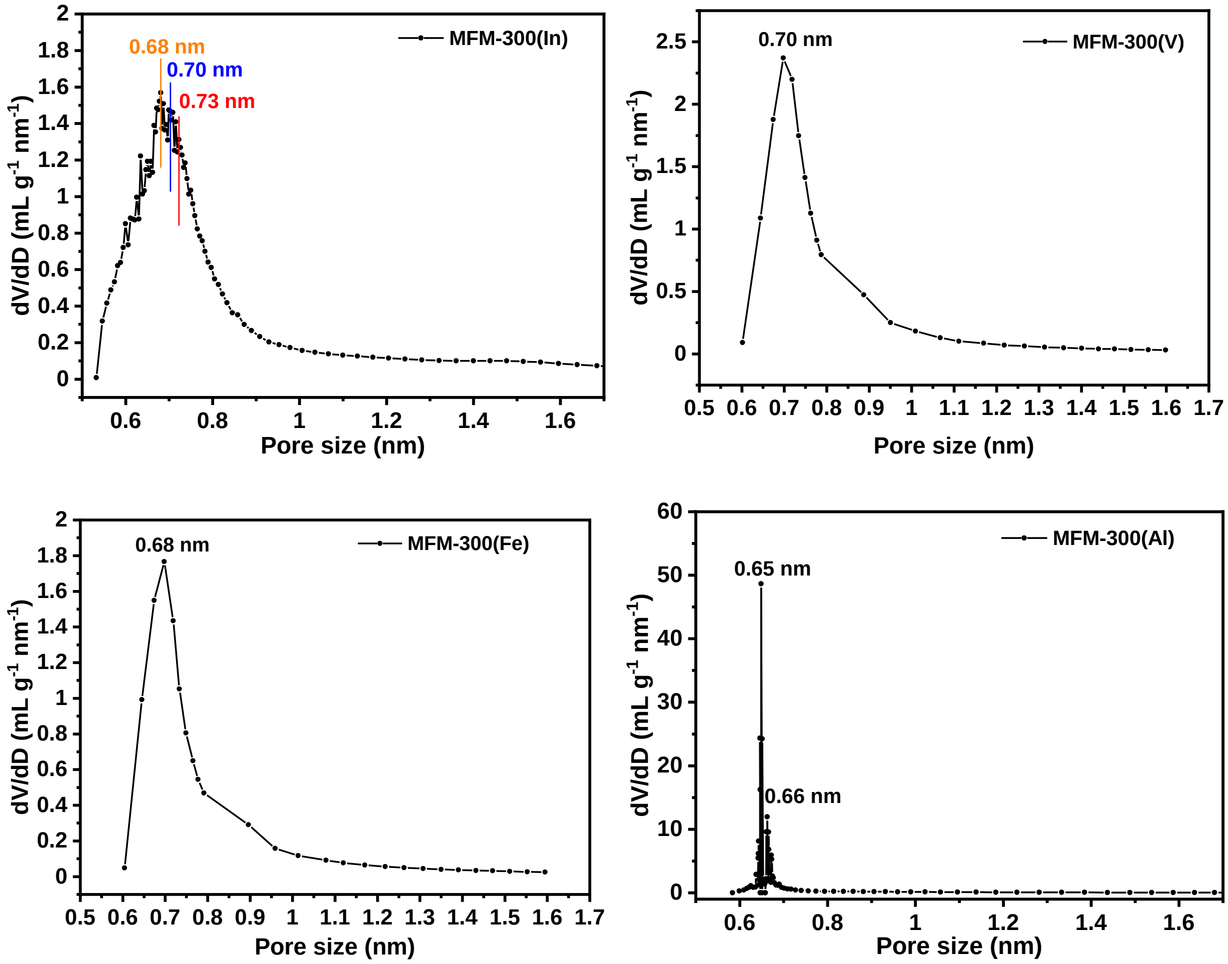

Supplementary Fig. 4. H-K pore size distribution for MFM-300(M) (M = In, Al) using data measured with N_2 (77K) and MFM-300(V and Fe) using data measured with CO_2 (273 K and 195 K, respectively).

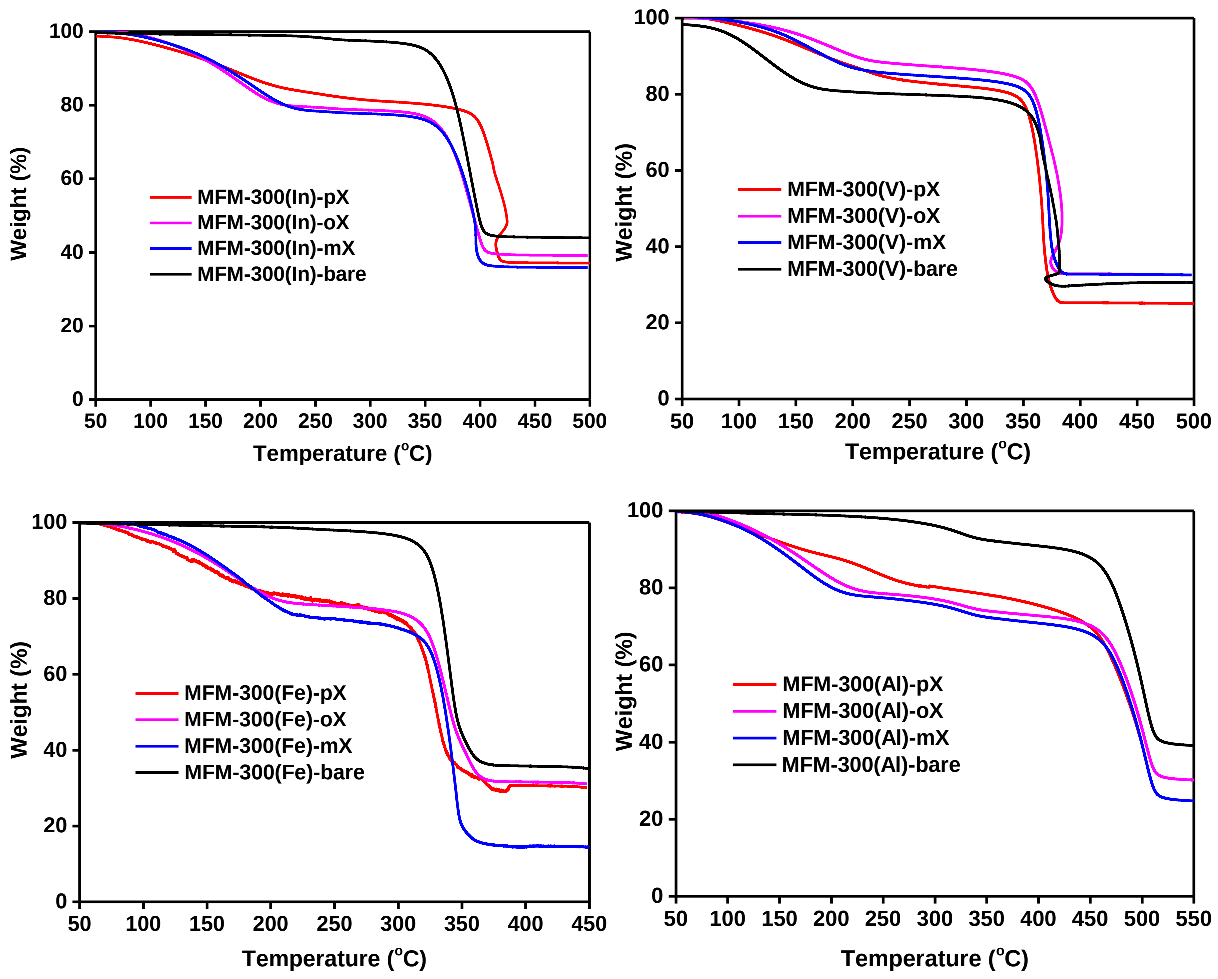

Supplementary Fig. 5. TGA of bare and xylene-loaded MFM-300(M) (M = In, V, Fe, Al).

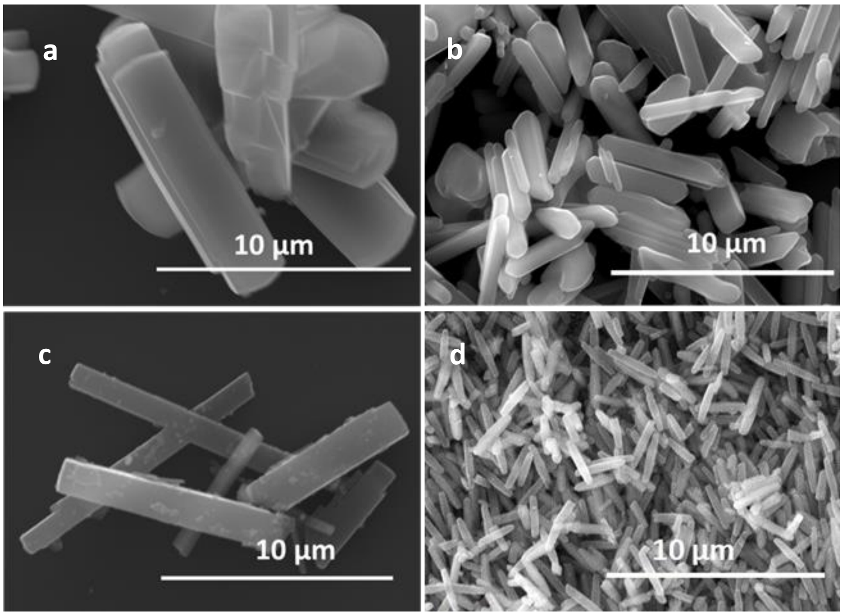

Supplementary Fig. 6. SEM images of **a,** MFM-300(In), **b,** MFM-300(V), **c,** MFM-300(Fe), **d,** MFM-300(Al). All particles exhibit a prismatic rod shape, and the average particle size of the as-synthesised MFM-300(M) (M = In, V, Fe, Al) is 10, 5, 12, and 2 µm, respectively.

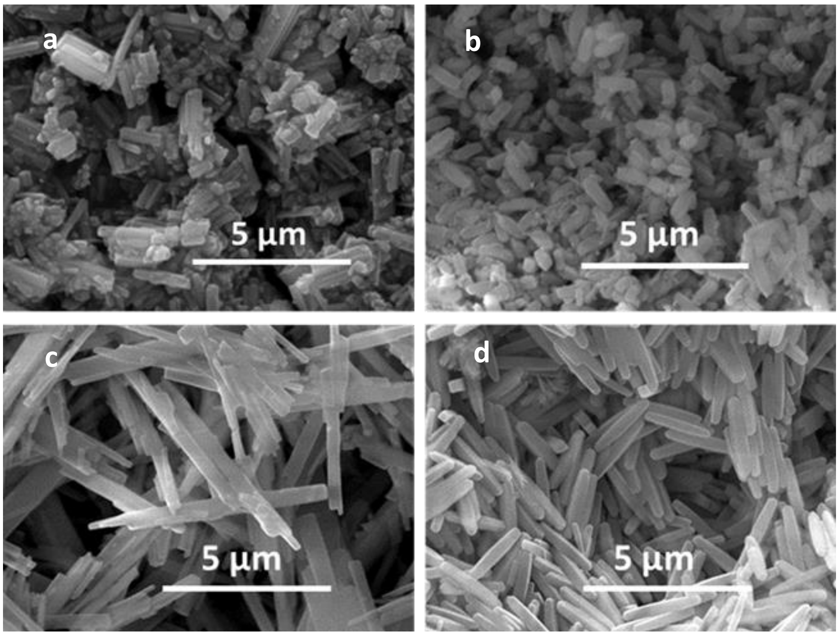

Supplementary Fig. 7. SEM images of ground MOF particles. **a,** MFM-300(In), **b,** MFM-300(V), **c,** MFM-300(Fe), **d,** MFM-300(Al). All particles are prismatic rods, and the average particle size of the ground MFM-300(M) (M = In, V, Fe, Al) is 5, 1, 5, and 2 µm, respectively.

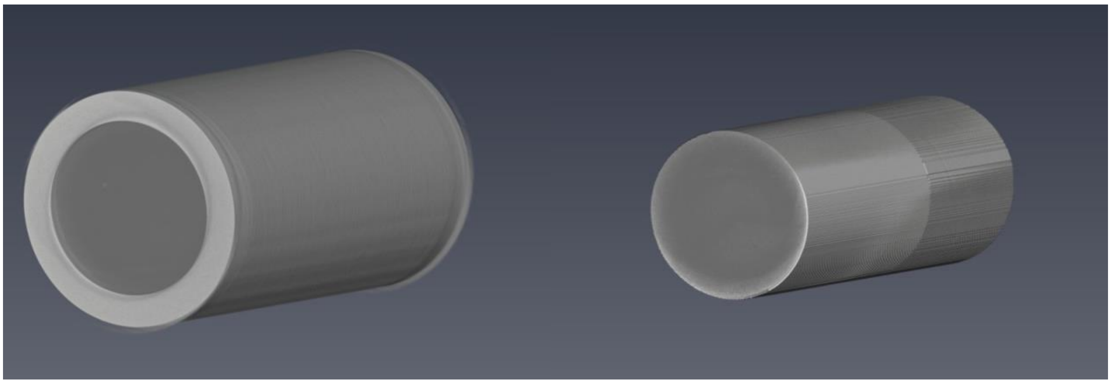

Supplementary Fig. 8. X-ray image of a column packed with MFM-300(In) (left) and of the MOF powder within the stainless steel shell (right). In the left picture, the light edge shell in the picture is the stainless steel column, and the dark area inside the column represents the MOF powder.

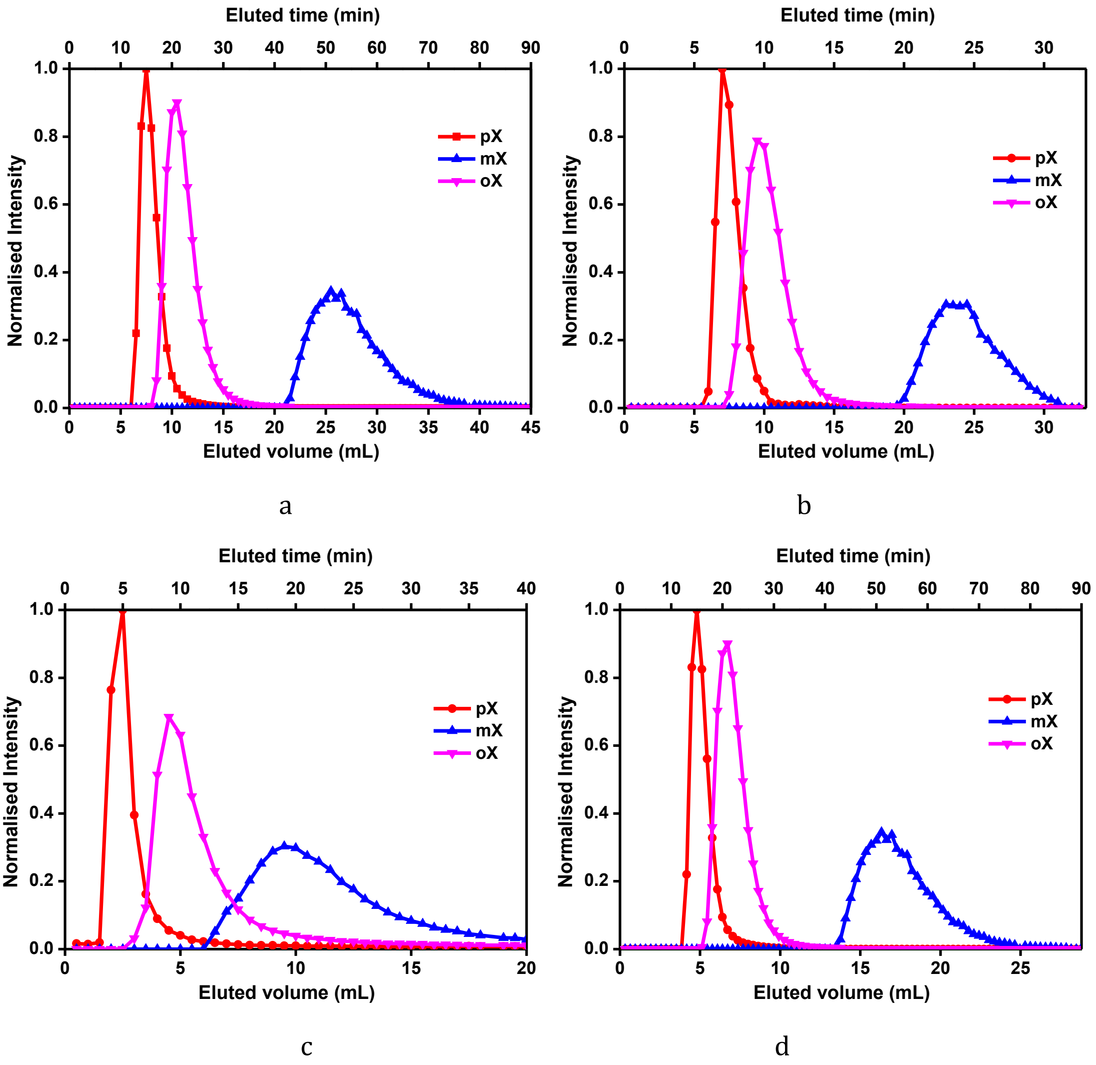

Supplementary Fig. 9. Effect of flow rate on xylene separation. All experiments were undertaken using *n*-pentane as eluent at 293K. **a,** xylene separation chromatogram of MFM-300(In) at a flow rate of 0.5 mL min^{-1}, **b,** xylene separation chromatogram of MFM-300(In) at a flow rate of 1.0 mL min^{-1}, **c,** xylene separation chromatogram of MFM-300(V) at a flow rate of 0.5 mL min^{-1}, **d,** xylene separation chromatogram of MFM-300(V) at a flow rate of 0.32 mL min^{-1}.

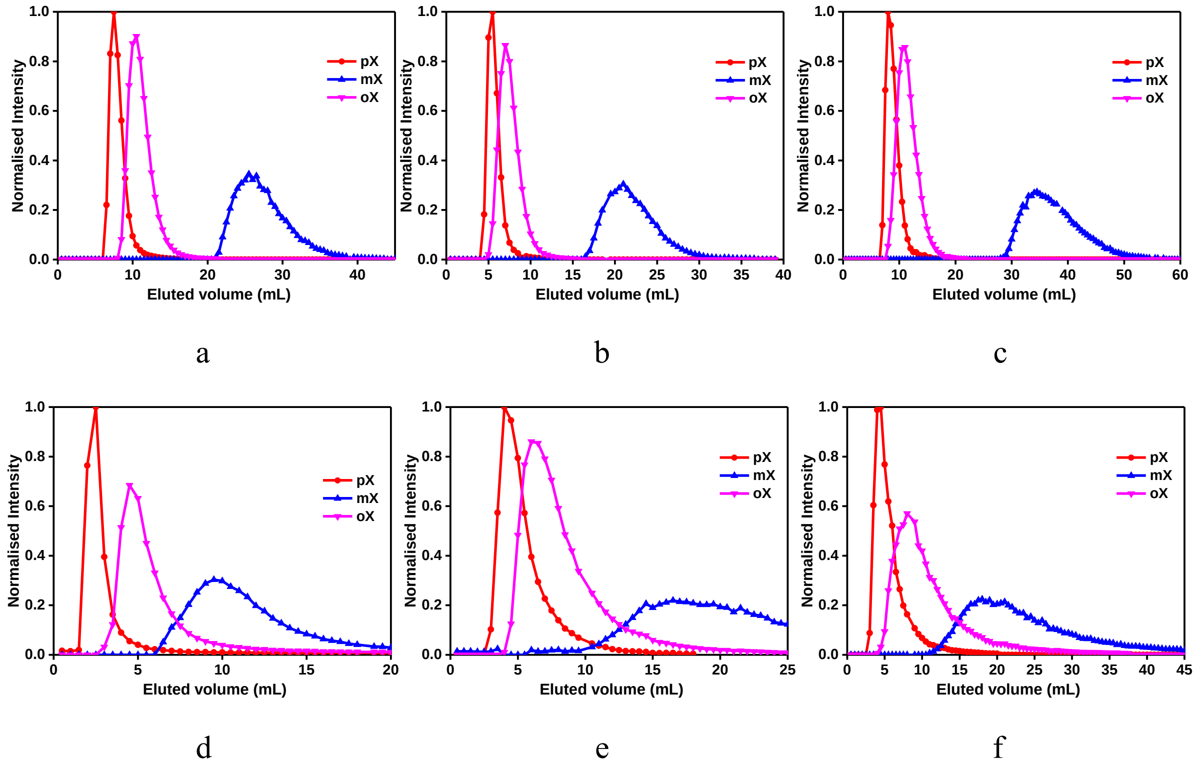

Supplementary Fig. 10. Effect of eluent on xylene separation. All experiments were undertaken at a flow rate of 0.5 mL min^{-1} and 293K. **a,** xylene separation chromatogram of MFM-300(In) using pentane as eluent, **b,** xylene separation chromatogram of MFM-300(In) using hexane as eluent, **c,** xylene separation chromatogram of MFM-300(In) using heptane as eluent, **d,** xylene separation chromatogram of MFM-300(V) using pentane as eluent, **e,** xylene separation chromatogram of MFM-300(V) using hexane as eluent, **f,** xylene separation chromatogram of MFM-300(V) using heptane as eluent. The choice of flow rate and eluent influences the observed adsorption selectivity. MFM-300(M) (M = In, V, Fe) presented a preference for *m*-xylene over *o*-xylene and *p*-xylene in all experiments.

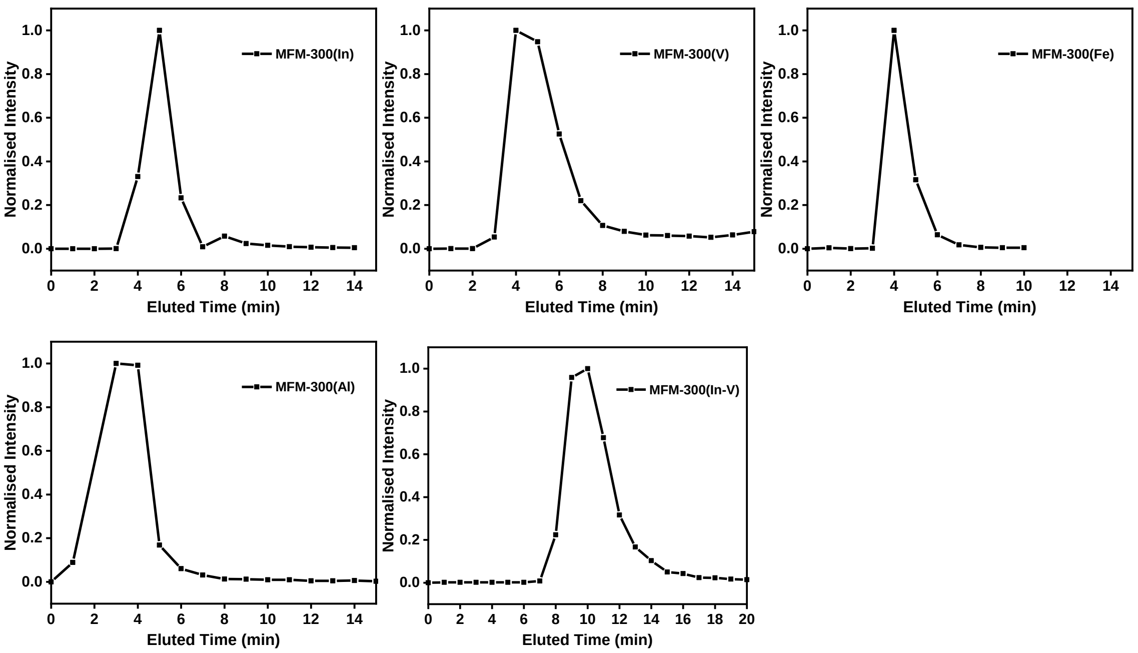

Supplementary Fig. 11. Bed void time tests using 1,3,5-triisopropylbenzene in different columns. Measurements were undertaken at 293 K.

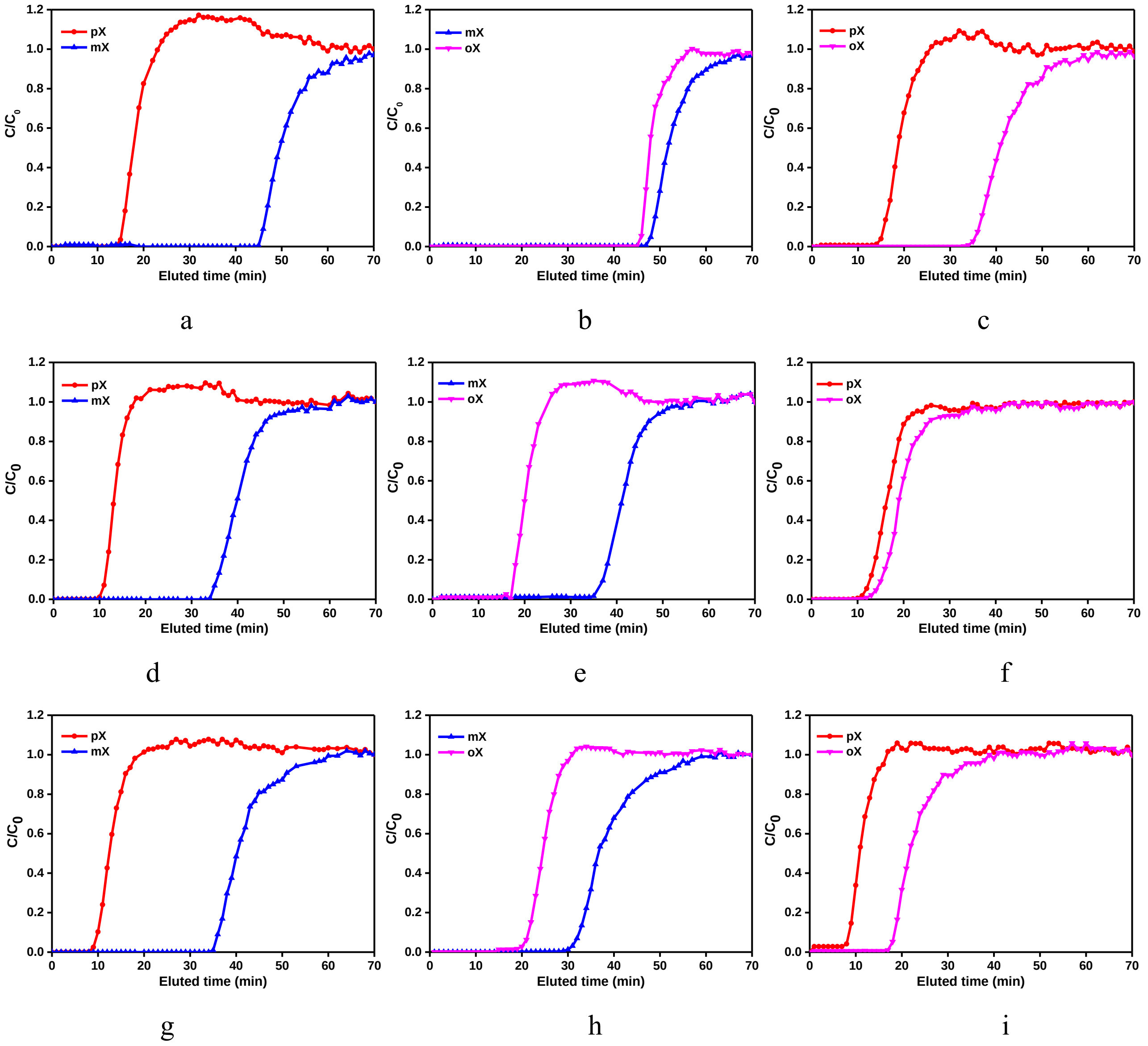

Supplementary Fig. 12. Binary breakthrough curves for equimolar xylene mixtures on **a-c,** MFM-300(V), **d-f**, MFM-300(In), **g-i,** MFM-300(Fe). Measurements were undertaken at 293 K.

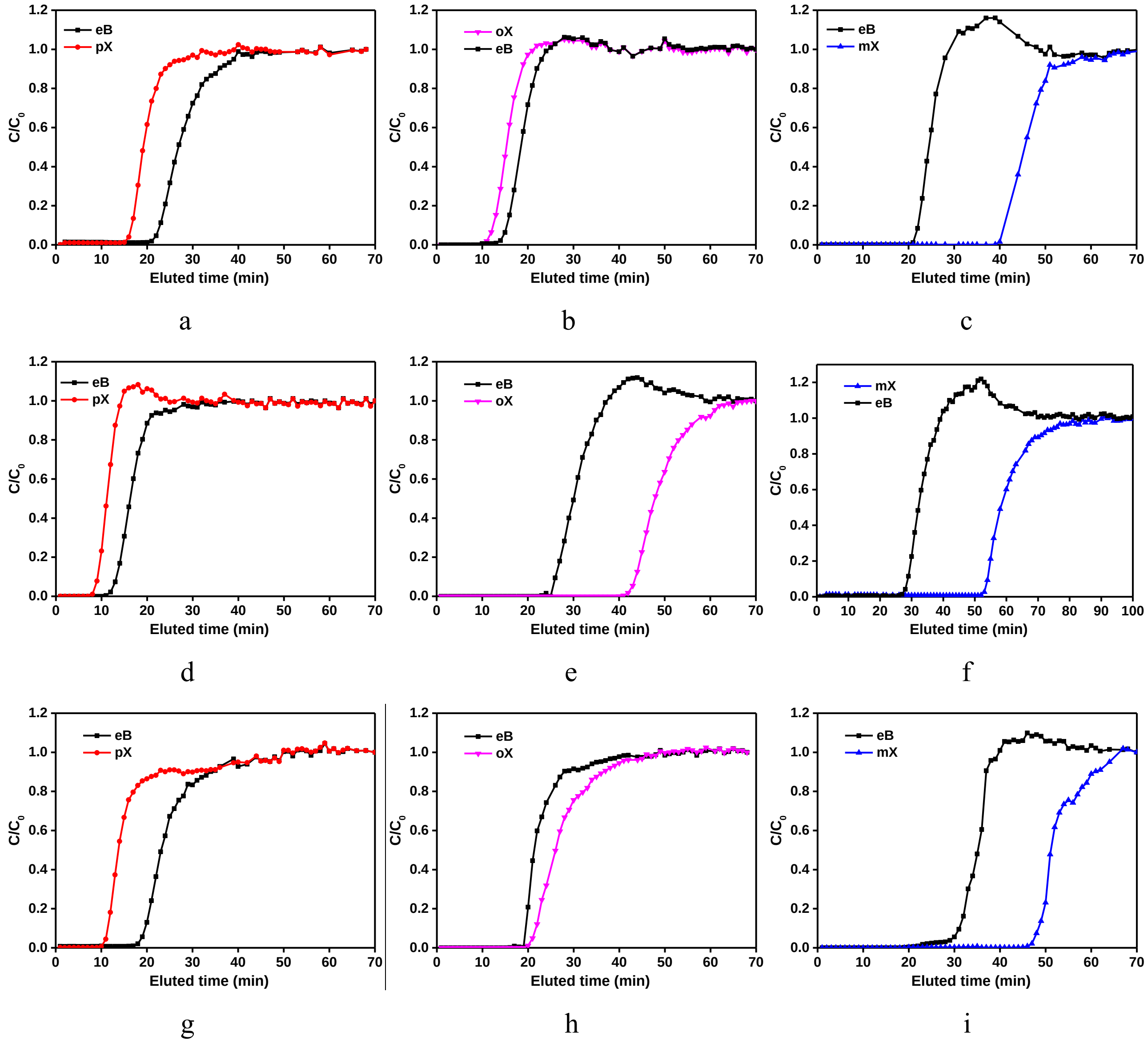

Supplementary Fig. 13. Binary breakthrough curves for equimolar xylene mixtures on **a-c,** MFM-300(In), **d-f**, MFM-300(V), **g-i,** MFM-300(Fe). Measurements were undertaken at 293 K.

Supplementary discussions of the "roll-up" effect

In a binary flow experiment once the column becomes saturated, the less preferred compound will elute first and the more preferred compound displaces the less preferred compound out of the pores into the effluent stream. This causes a temporary increase in the outlet concentration of the least preferred compound. As expected, this indicates that the xylene isomers compete for the same space within the pores of the MOF[8]. In all of the materials, the roll-up effect is synchronous with the breakthrough of *m*-xylene, indicating that the more strongly adsorbing *m*-xylene displaces the initially adsorbed *p*-xylene and *o*-xylene from the MFM-300 pores. Compared to MFM-300(M) (M = In, Fe), the experimental results point to MFM-300(V) having the greatest roll-up effect for *p*-xylene and *o*-xylene. This illustrates that small alterations in pore size can strongly influence the adsorption and separation of these organic substrates.

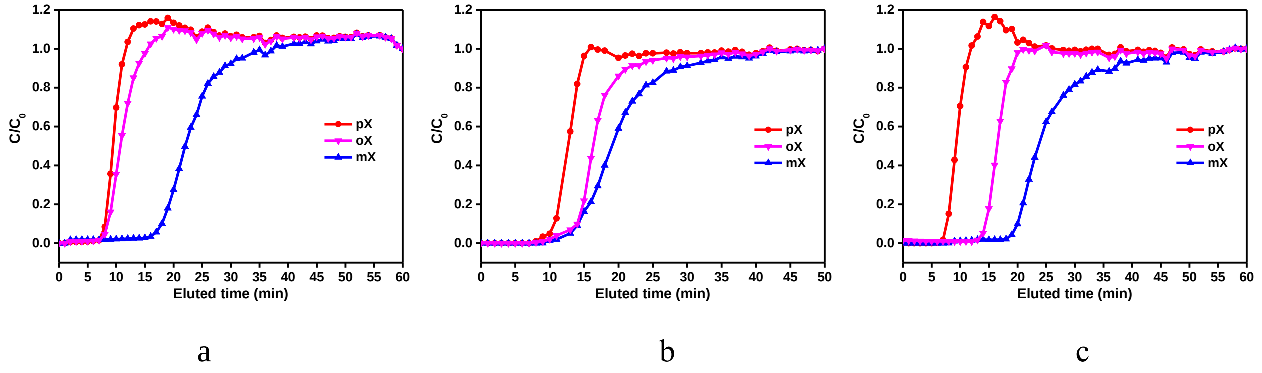

Supplementary Fig. 14. Ternary breakthrough curves of **a,** MFM-300(In), **b,** MFM-300(V), **c,** MFM-300(Fe). The breakthrough curves were carried out by pumping an equimolar xylene mixture solution (50mM) into the column and collecting samples every 1min. Measurements were undertaken at 293 K.

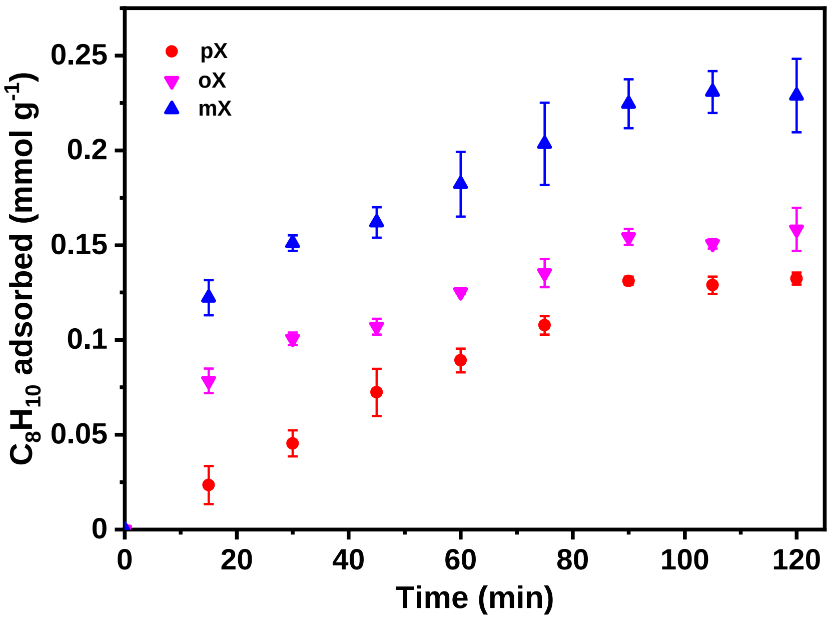

Supplementary Fig. 15. Single-component liquid-phase adsorption kinetics of xylenes at 293 K in MFM-300(In) using 15mM solutions in *n*-pentane: *p*-xylene (red), *o*-xylene (magenta), *m*-xylene (blue).

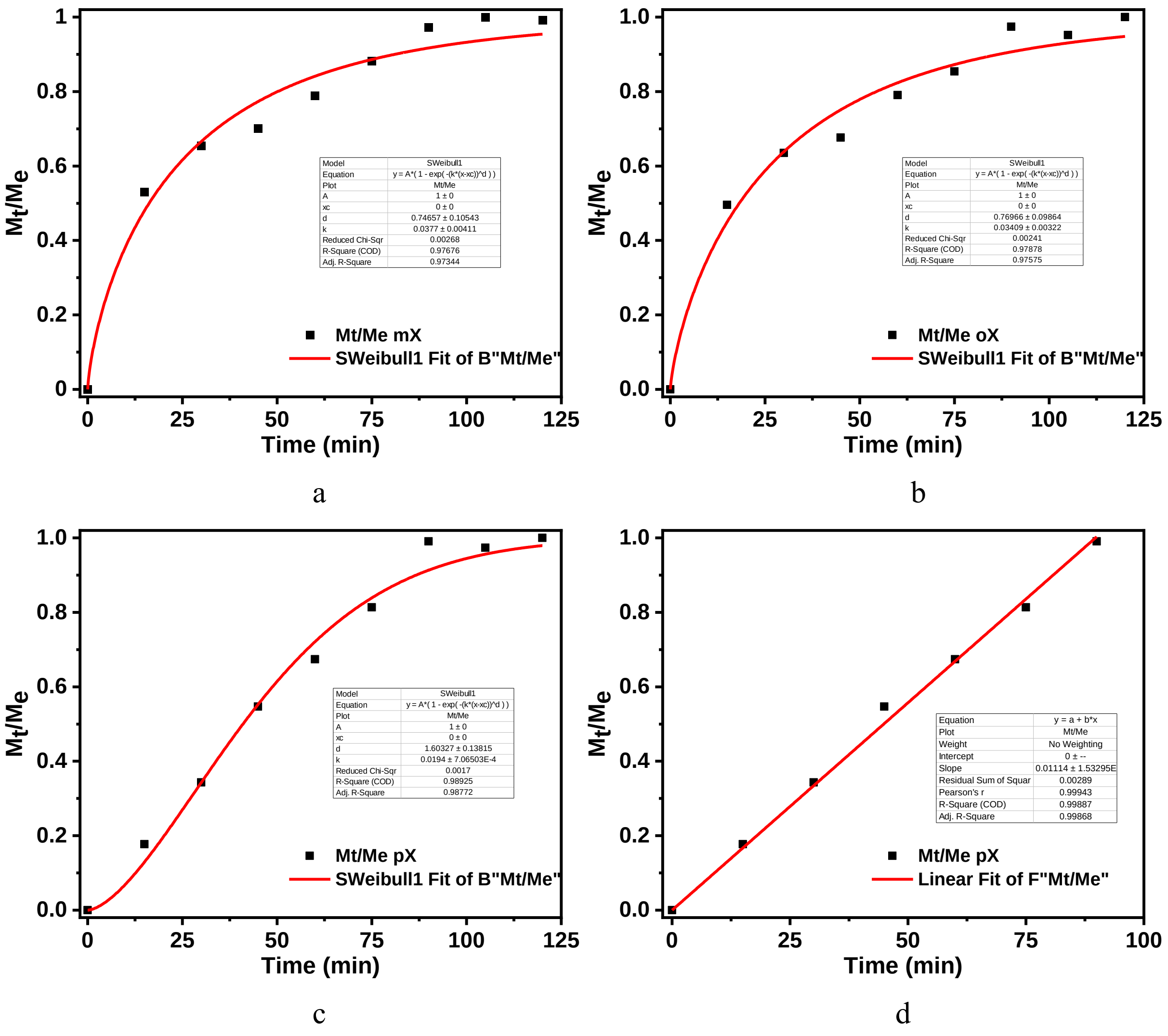

Supplementary Fig. 16. Fitting of the stretched exponential model to the normalized kinetic profiles of **a**, *m*-xylene, **b**, *o*-xylene, **c**, *p*-xylene. **d**. Plot of M_t/M_e versus time based upon liquid-phase adsorption kinetics curves for *p*-xylene.

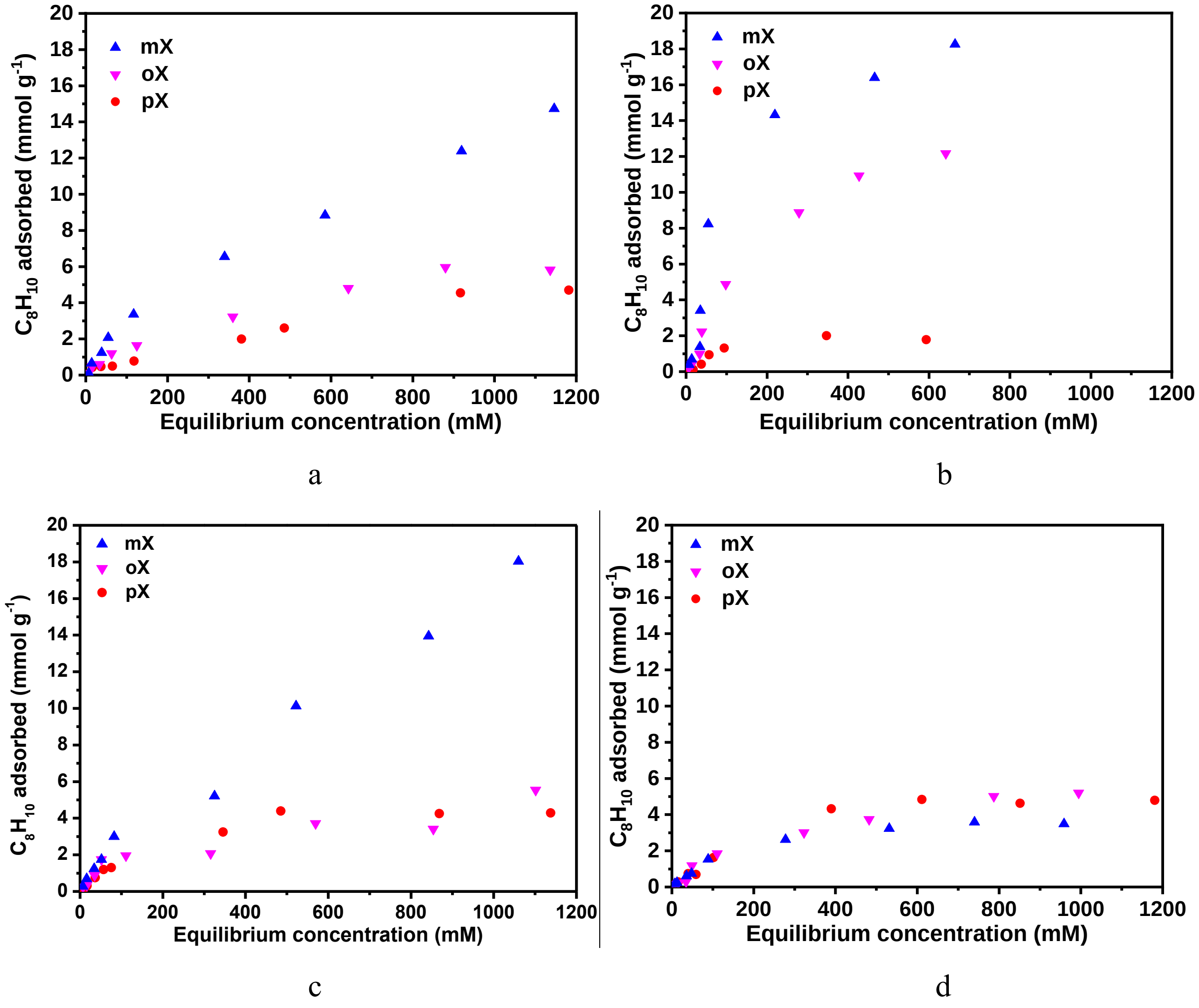

Supplementary Fig. 17. Single-component liquid-phase adsorption measurements for xylenes in **a,** MFM-300(In), **b,** MFM-300(V), **c,** MFM-300(Fe) and **d,** MFM-300(Al) at 293K in *n*-pentane: *p*-xylene (red), *o*-xylene (magenta), *m*-xylene (blue).

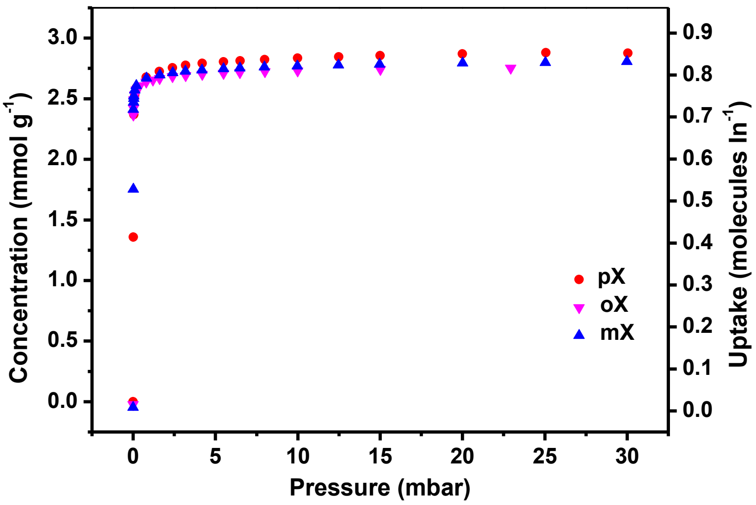

a

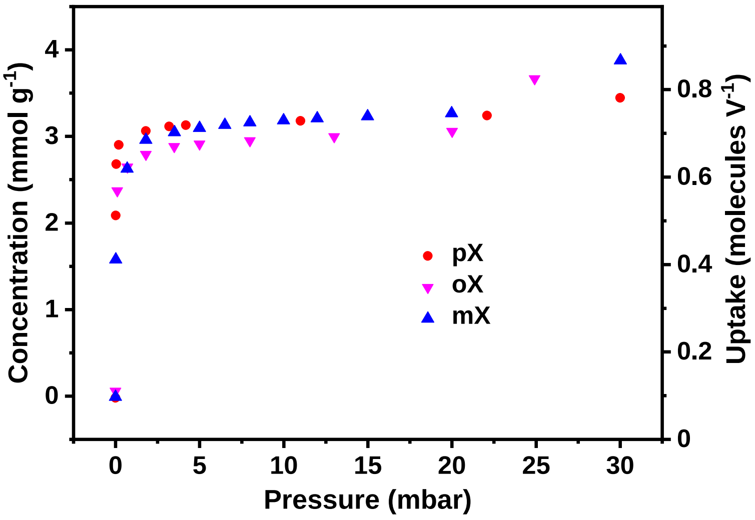

b

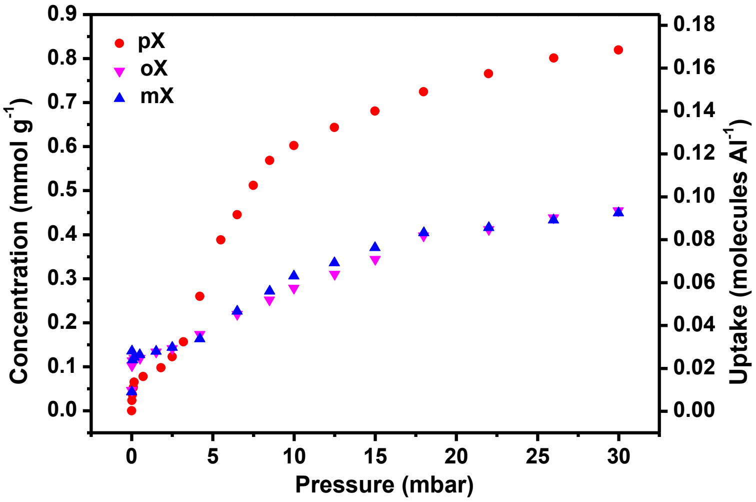

c

Supplementary Fig. 18. Adsorption isotherms for xylene vapours in **a,** MFM-300(In), **b,** MFM-300(V) and **c,** MFM-300(Al) recorded at 318K.

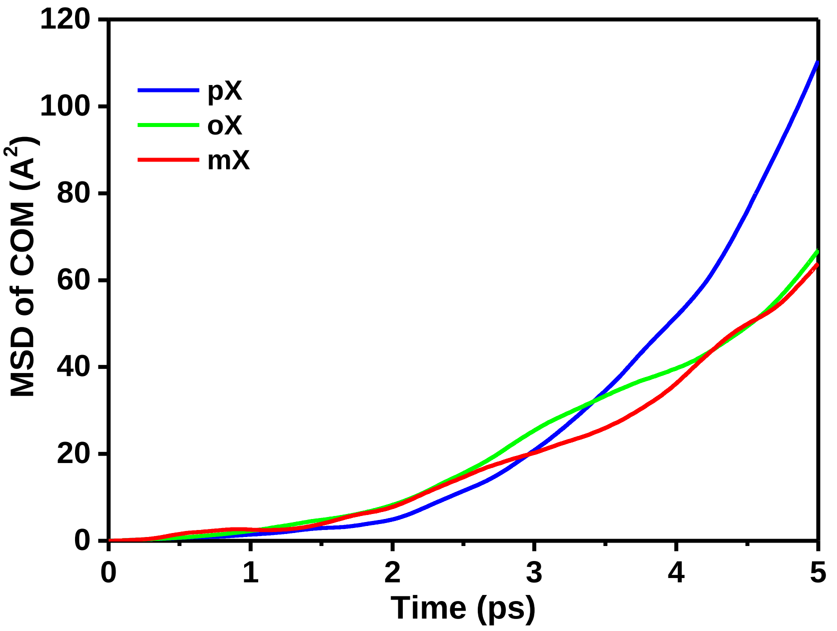

Supplementary Fig. 19. Simulation of the mean square displacement of the xylene molecules diffusing inside MFM-300(In) under a constant driving force. The mobility of *p*-xylene is significantly higher than that of *m*-xylene and *o*-xylene. See Methods Section for details of the simulation.

a

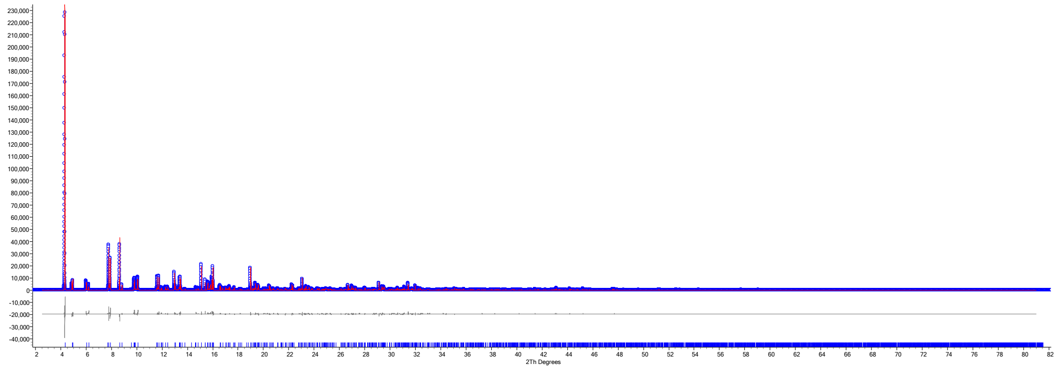
Counts
2Th Degrees

b

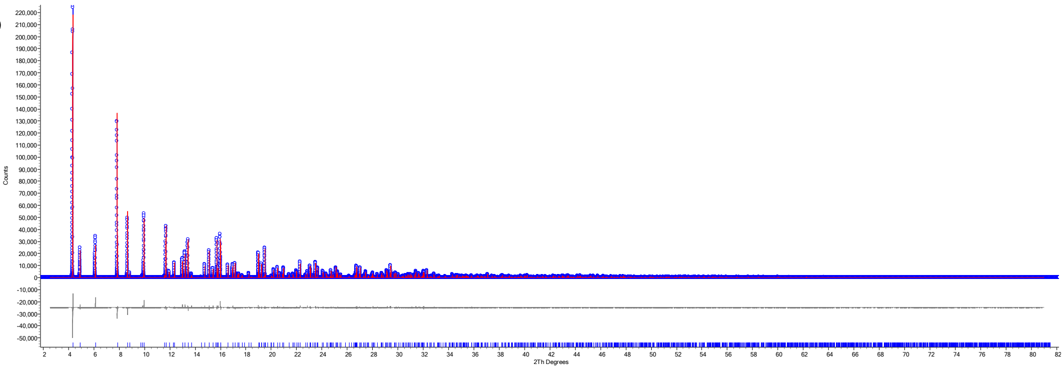
Counts
2Th Degrees

c

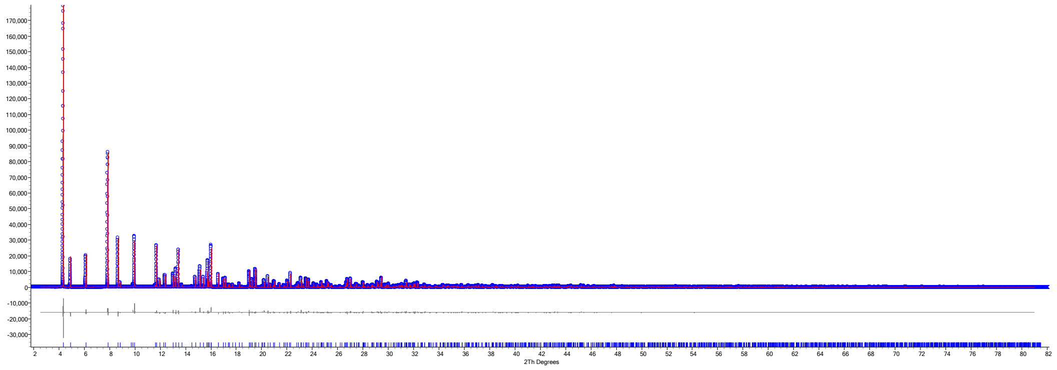
Counts
2Th Degrees

d

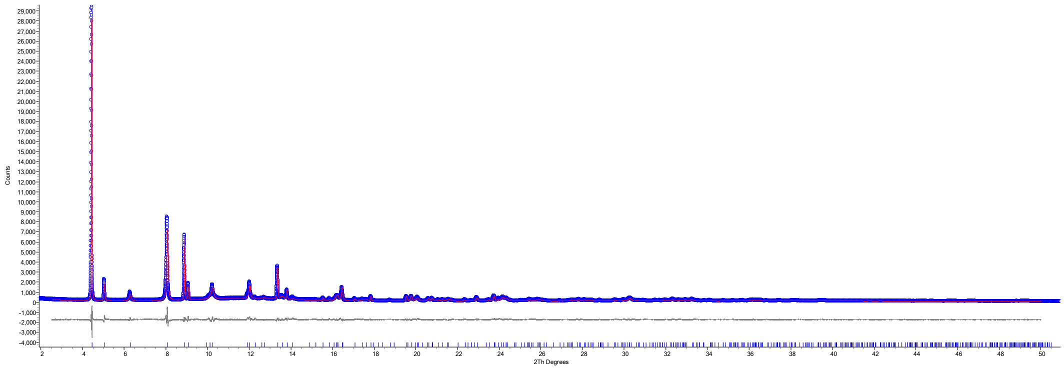
Counts
2Th Degrees

e

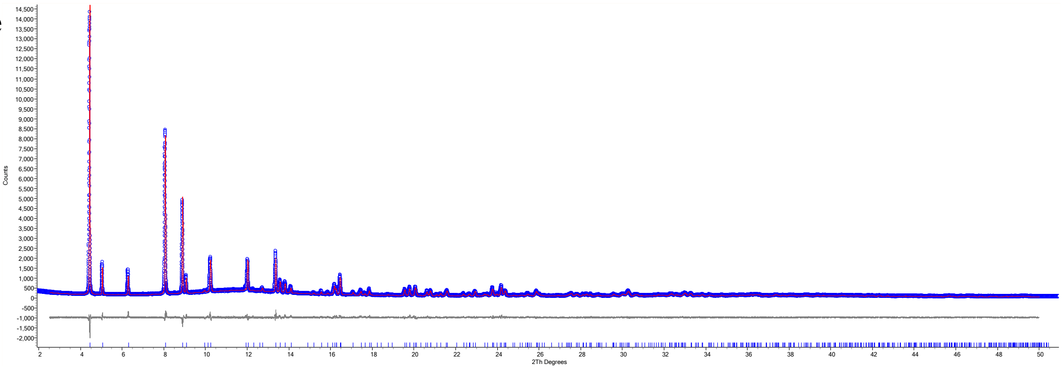
Counts
2Th Degrees

f

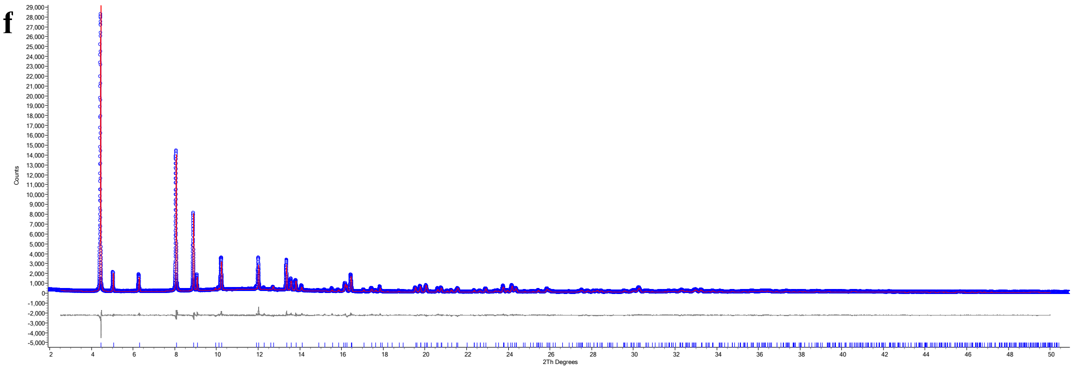
Counts
2Th Degrees

g

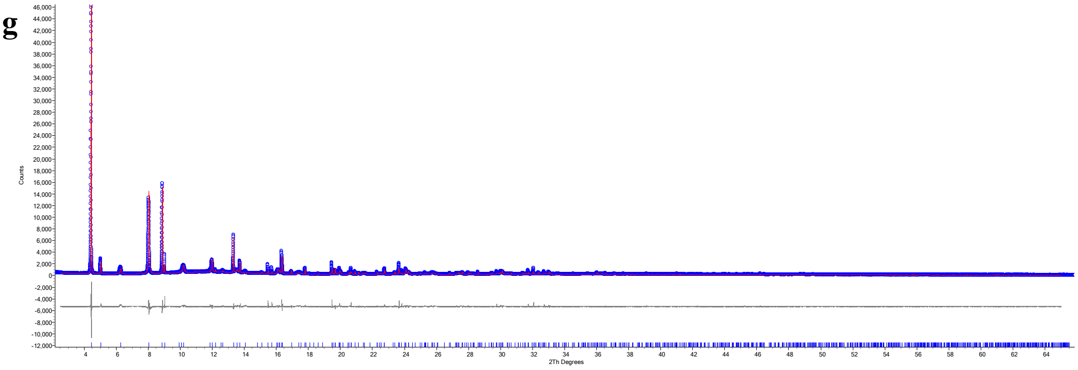
Counts
2Th Degrees

h

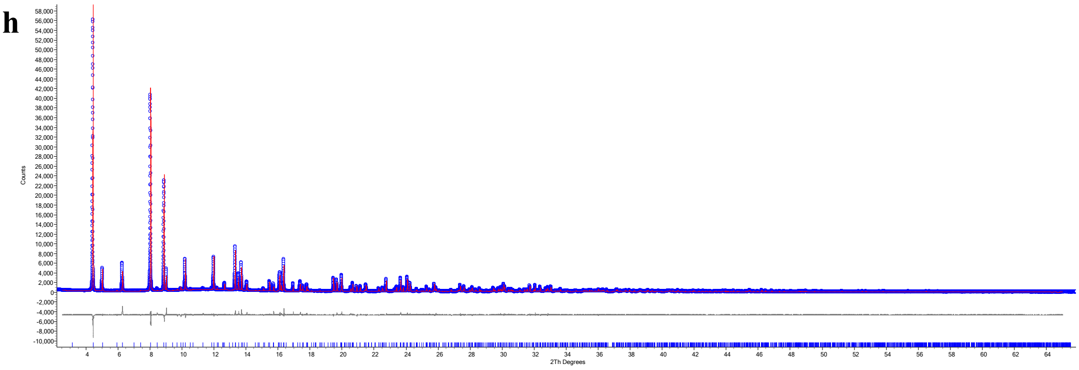
Counts
2Th Degrees

i

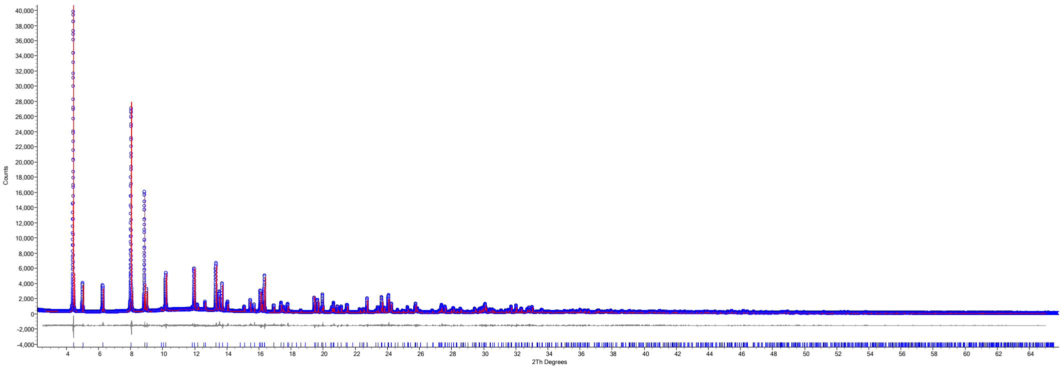
Counts
2Th Degrees

j

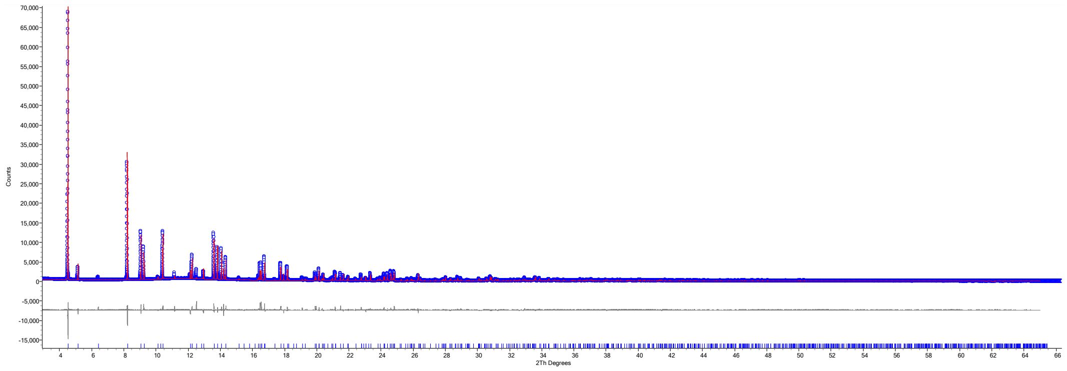

k

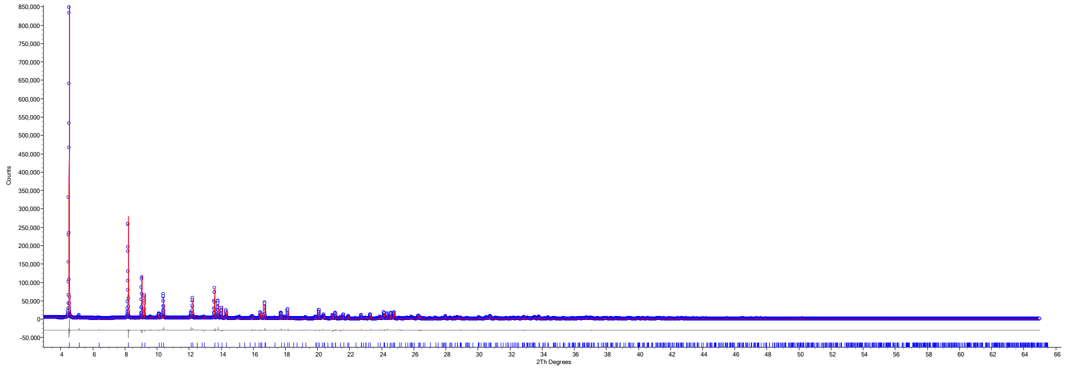

l

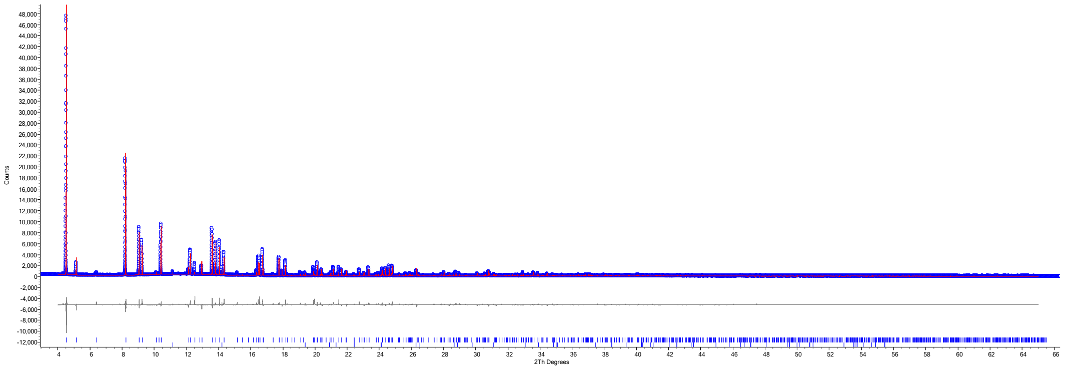

Supplementary Fig. 20. Rietveld refinement for *p*-, *o*-, *m*-xylene-loaded **a-c,** MFM-300(In), **d-f,** MFM-300(V), **g-i,** MFM-300(Fe), **j-l**, MFM-300(Al) from PXRD data measured at room temperature. Blue points are experimental data and the red line represents the fitting.

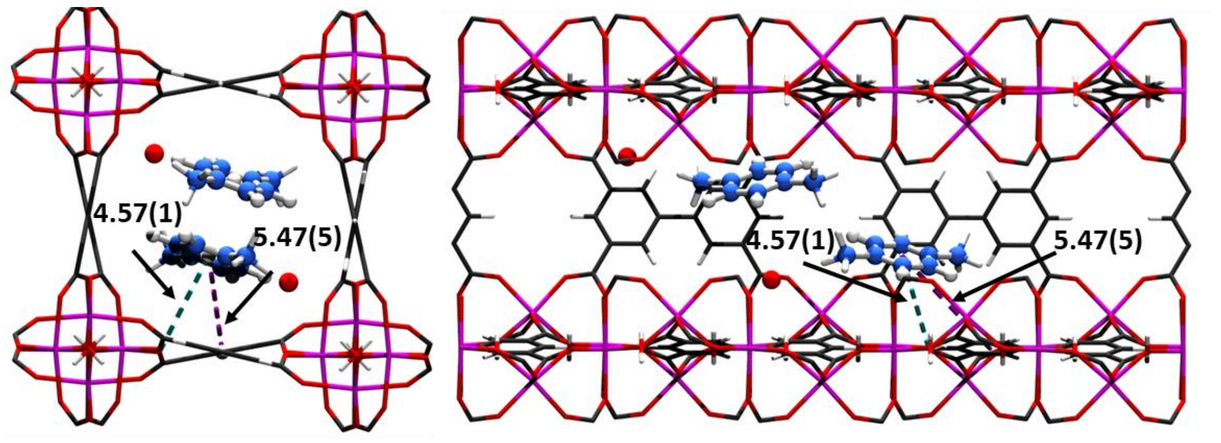

a

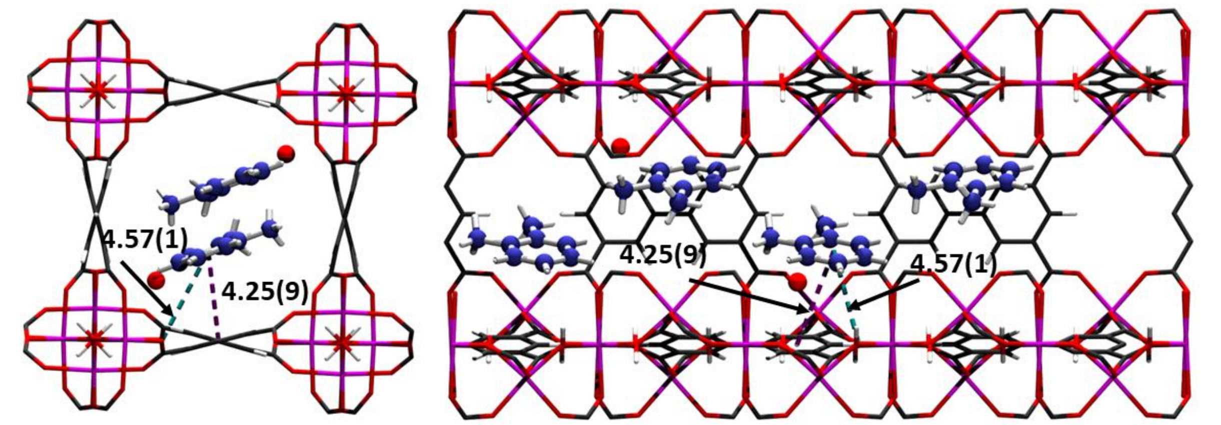

b

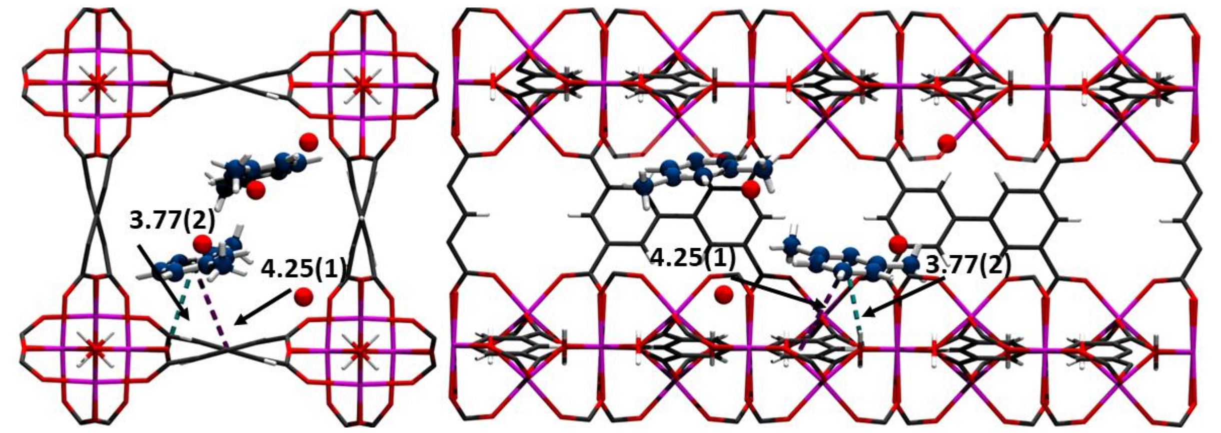

c

Supplementary Fig. 21. Refined positions for xylenes in MFM-300(In). **a,** Views of the structure of MFM-300(In)[*p*-xylene]$_{1.00}$ along the *c* axis (left) and the *a* axis(right), **b,** views of the structure of MFM-300(In)[*o*-xylene]$_{0.50}$ along the *c* axis(left) and the *a* axis(right), **c,** views of the structure of MFM-300(In)[*m*-xylene]$_{0.50}$ along the *c* axis(left) and the *a* axis(right). The structure was solved from high-resolution PXRD data. The hydrogen atoms of xylene molecules have been omitted for clarity

(indium, magneta; carbon, grey; oxygen, red; hydrogen, white). The π···π interactions between aromatic rings of xylene molecules and L^{4-} ligands are highlighted as violet dashed lines with distances of 5.47(5), 4.25(9) and 4.25(1) Å for *p*-xylene, *o*-xylene, *m*-xylene, respectively. The relatively strong π···O interactions between the phenyl ring of xylene molecules and oxygen atoms of carboxylate groups in linkers are highlighted in teal, with distances of 4.57(0) Å, 4.70(1), 3.77(2) for *p*-xylene, *o*-xylene, *m*-xylene, respectively. The occupancies were determined as 0.50, 0.25, and 0.25 for *p*-xylene, *o*-xylene and *m*-xylene, respectively.

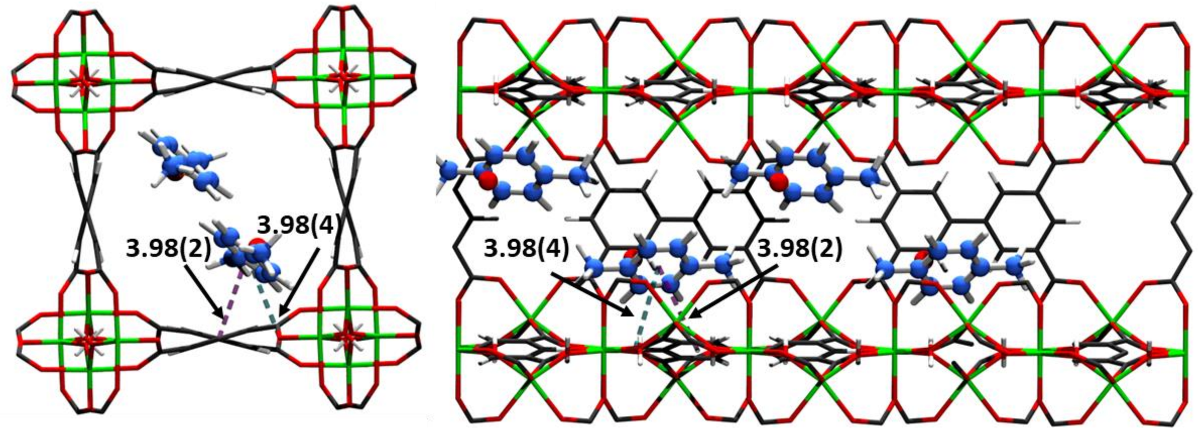

a

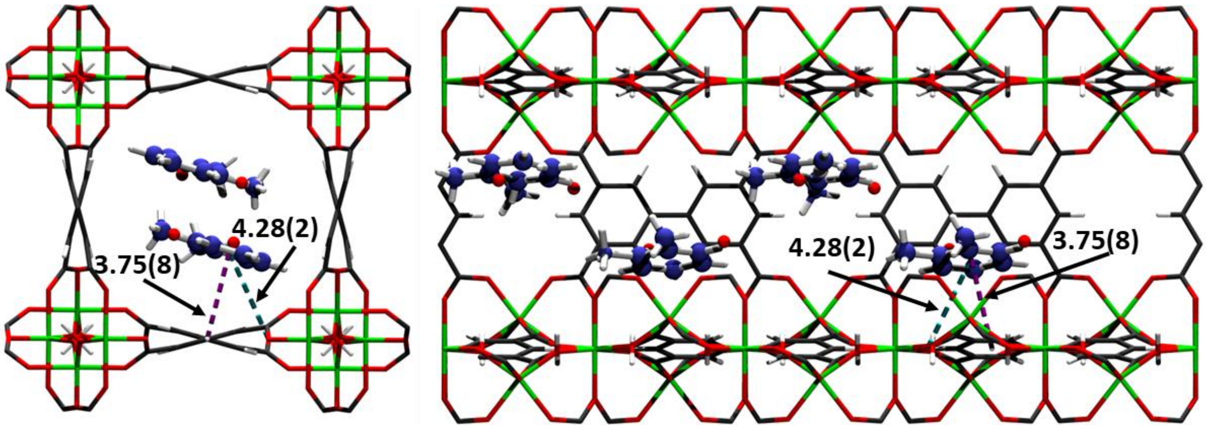

b

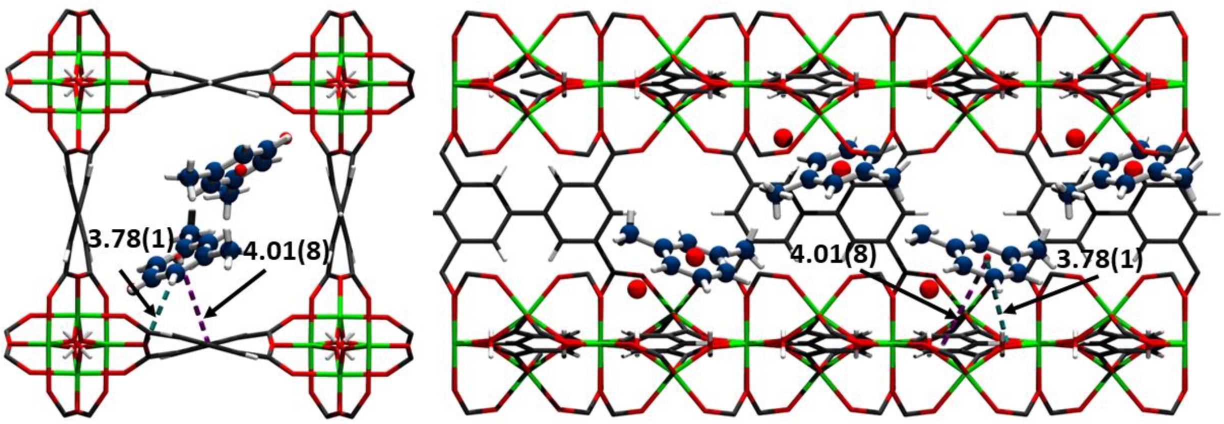

c

Supplementary Fig. 22. Refined positions for xylene in MFM-300(V). **a,** Views of the structure of MFM-300(V)[*p*-xylene]$_{0.50}$ along the *c* axis(left) and the *a* axis (right), **b,** views of the structure of MFM-300(V)[*o*-xylene]$_{0.50}$ along the *c* axis (left) and the *a* axis (right) , **c,** views of the structure of MFM-300(In)[*m*-xylene]$_{0.50}$ along the *c* axis (left) and the *a* axis (right). The structure was solved

from high-resolution PXRD data. The hydrogen atoms of xylene molecules have been omitted for clarity (vanadium, green; carbon, grey; oxygen, red; hydrogen, white). The π···π interactions between aromatic rings of xylene molecules and L^{4-} ligands are highlighted by violet dashed lines. The distances are 3.98(2), 3.75(8), 4.01(8) Å for *p*-, *o*- and *m*-xylene, respectively. The relatively strong π···O interactions between the phenyl ring of xylene molecules and oxygen atoms of carboxylic groups in linkers are highlighted in teal, with distances of 3.98(4) Å, 4.28(2) Å, 3.78(0) Å for *p*-, *o*- and *m*-xylene, respectively. The occupancies were determined as 0.25, 0.25, and 0.25 for *p*-, *o*- and *m*-xylene, respectively.

Only host-guest π···π interactions for *p*- and *o*-xylene-loaded structures of MFM-300(Fe) are observed at distances of 4.12(2) Å and 3.79(8) Å, respectively (Supplementary Figs. 22 and 23). MFM-300(Fe) shows a phase change from $I4_122$ to $P4_122$ when loaded with *o*-xylene. In *m*-xylene-loaded MFM-300(Fe), the distance for the host-guest π···π interaction is 3.73(9) Å (Supplementary Fig. 25). Therefore, *m*-xylene is the most tightly-held molecule of the three xylene isomers due to the strong cooperative binding interactions within the pore.

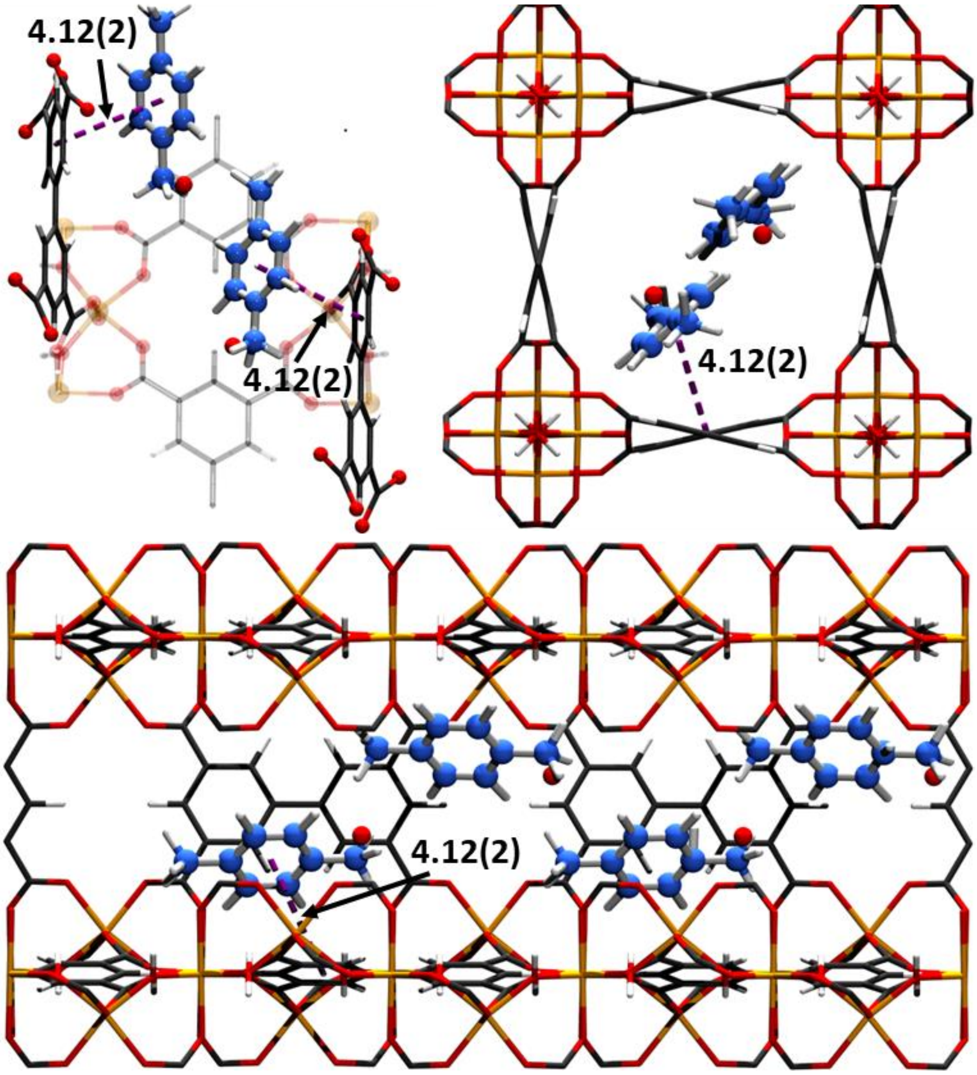

Supplementary Fig. 23. Refined positions for *p*-xylene in MFM-300(Fe) and views of the structure of MFM-300(Fe)[*p*-xylene]$_{0.50}$. The structure was solved from high-resolution PXRD data. The hydrogen atoms of xylene molecules have been omitted for clarity (iron, teal; carbon, grey; oxygen, red; hydrogen, white). The π···π interactions between aromatic rings of the xylene molecules and L^{4-} ligands are highlighted in violet dashed lines with a distance of 4.12(2) Å. The occupancy was determined as 0.25.

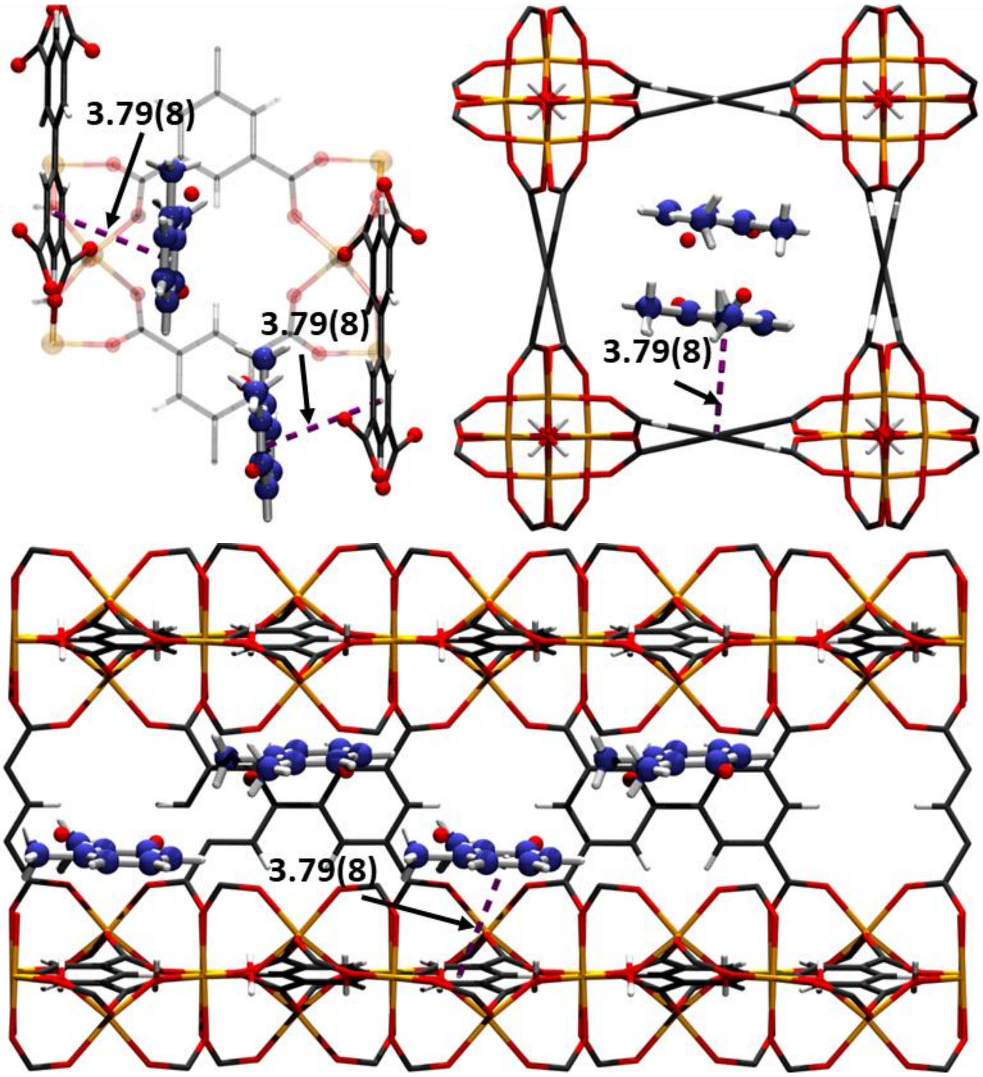

Supplementary Fig. 24. Refined positions for *o*-xylene in MFM-300(Fe) and views of the structure of MFM-300(Fe)[*o*-xylene]$_{0.50}$. The structure was solved from high-resolution PXRD data. The hydrogen atoms of xylene molecules have been omitted for clarity (iron, teal; carbon, grey; oxygen, red; hydrogen, white). The π···π interactions between aromatic rings of xylene molecules and L^{4-} ligands are highlighted in violet dashed lines with a distance of 3.79(8) Å. The occupancy is 0.25.

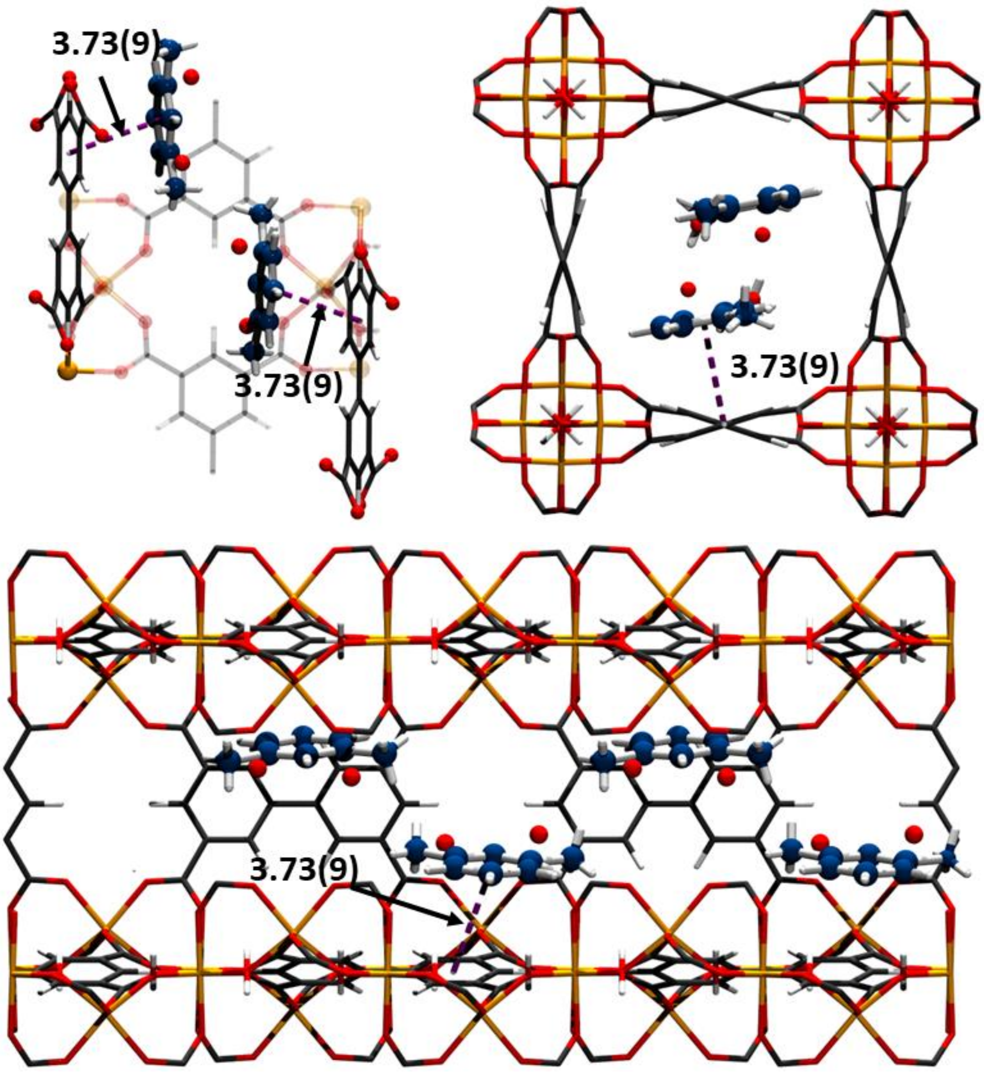

Supplementary Fig. 25. Refined positions for *m*-xylene in MFM-300(Fe) and views of the structure of MFM-300(Fe)[*m*-xylene]$_{0.50}$. The structure was solved from high-resolution PXRD data. The hydrogen atoms of the xylene molecules have been omitted for clarity (iron, teal; carbon, grey; oxygen, red; hydrogen, white). The π···π interactions between aromatic rings of xylene molecules and L^{4-} ligands are highlighted in violet dashed lines with a distance of 3.73(9) Å. The occupancy was determined as 0.25.

For MFM-300(Al), the π···π interactions between aromatic rings of xylene molecules and ligands are highlighted as violet dashed lines; the distances are similar for all three xylene isomers at 3.74(2), 3.53(5) and 3.53(8) Å for *p*-, *o*-, *m*- xylene, respectively. The occupancy was determined as 0.25, 0.25, and 0.25 for *p*-, *o*- and *m*-xylene, respectively. This is consistent as there is not much difference in the elution time of three xylene isomers.

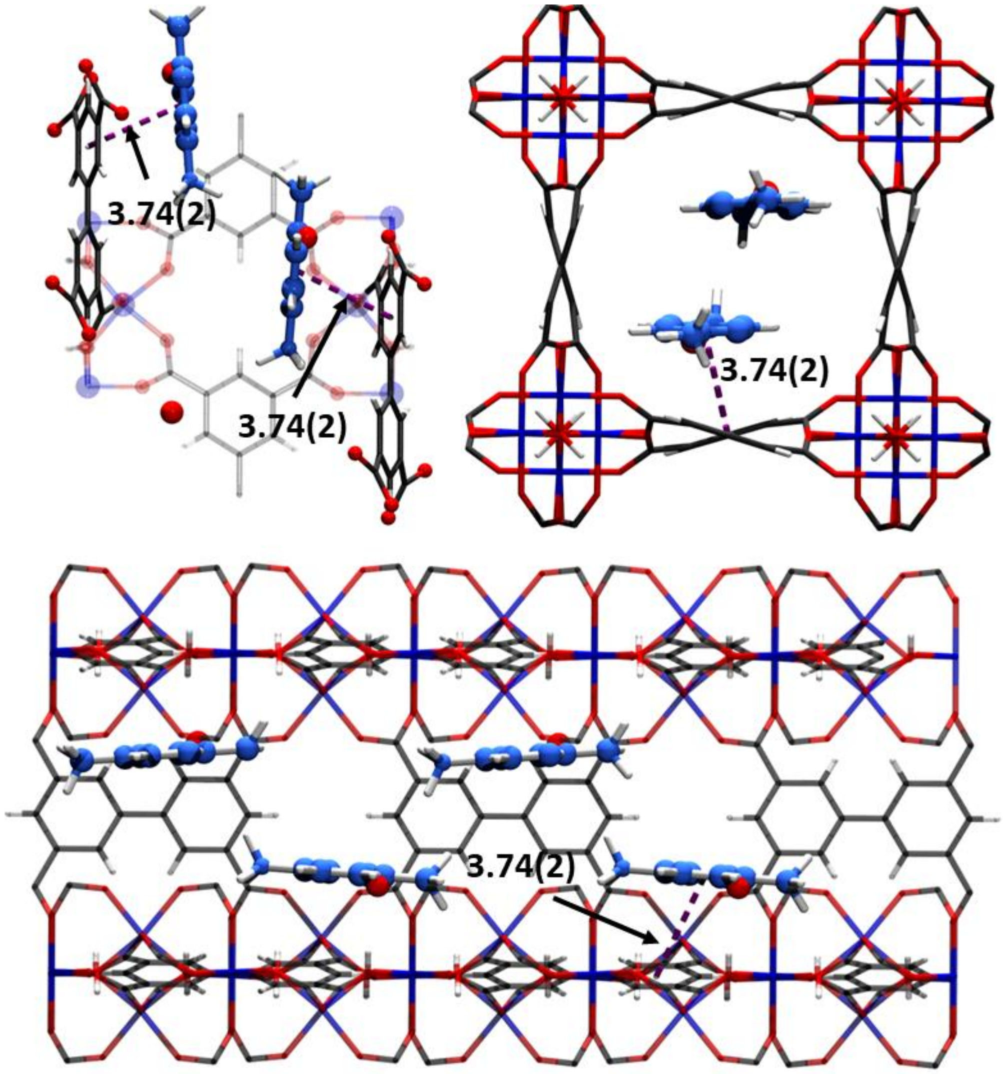

Supplementary Fig. 26. Refined positions of *p*-xylene in MFM-300(Al) and three views of the structure of MFM-300(Al)[*p*-xylene]$_{0.50}$. The structure was solved from high-resolution PXRD data. The hydrogen atoms of xylene molecules have been omitted for clarity (aluminium, blue; carbon, grey; oxygen, red; hydrogen, white). The π···π interactions between aromatic rings of xylene molecules and ligands L^{4-} are highlighted in violet dashed lines; the distance is 3.74(2) Å. The occupancy is determined as 0.25.

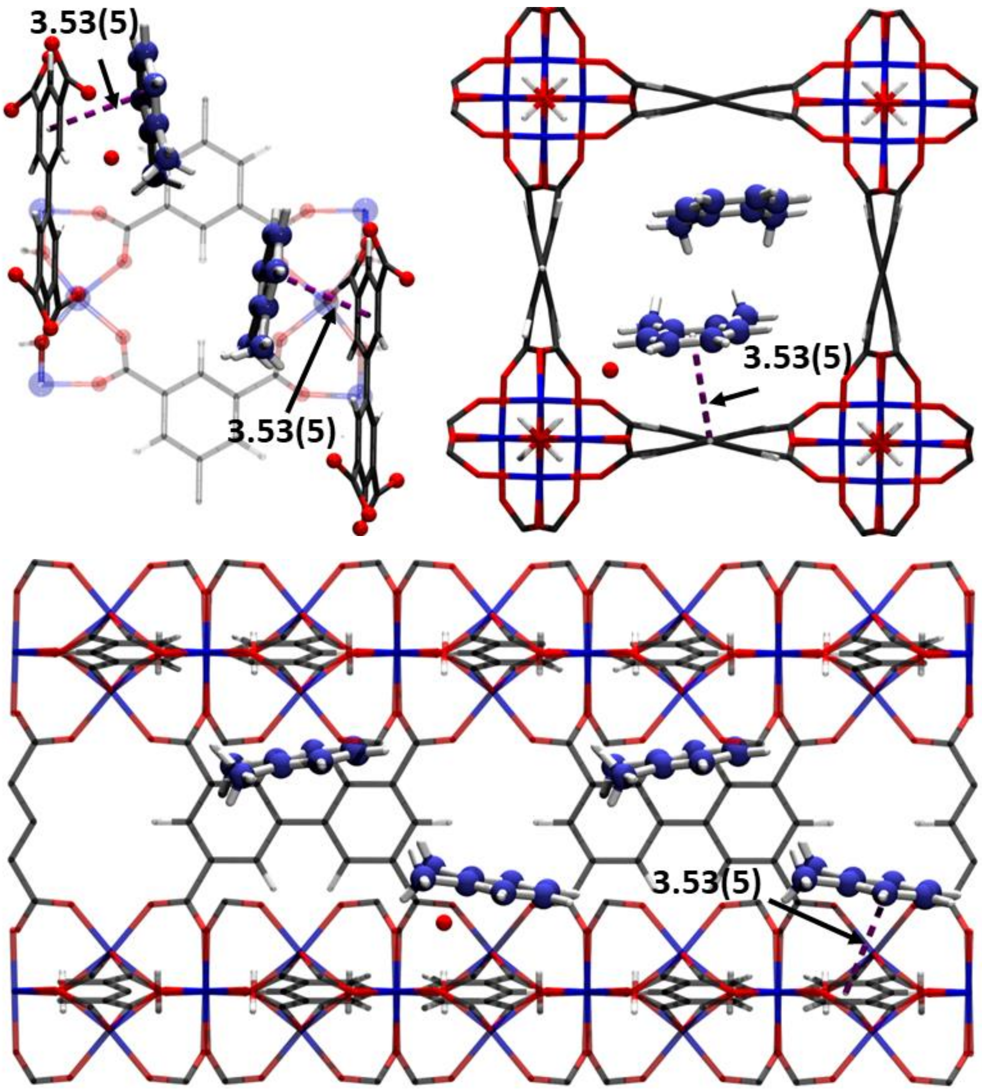

Supplementary Fig. 27. Refined positions for *o*-xylene in MFM-300(Al) and three views of the structure of MFM-300(Al)[*o*-xylene]$_{0.50}$. The structure was solved from high-resolution PXRD data. The hydrogen atoms of xylene molecules have been omitted for clarity (aluminium, blue; carbon, grey; oxygen, red; hydrogen, white). The π···π interactions between aromatic rings of xylene molecules and ligands are highlighted in violet dashed lines; the distance is 3.53(5) Å. The occupancy is 0.25.

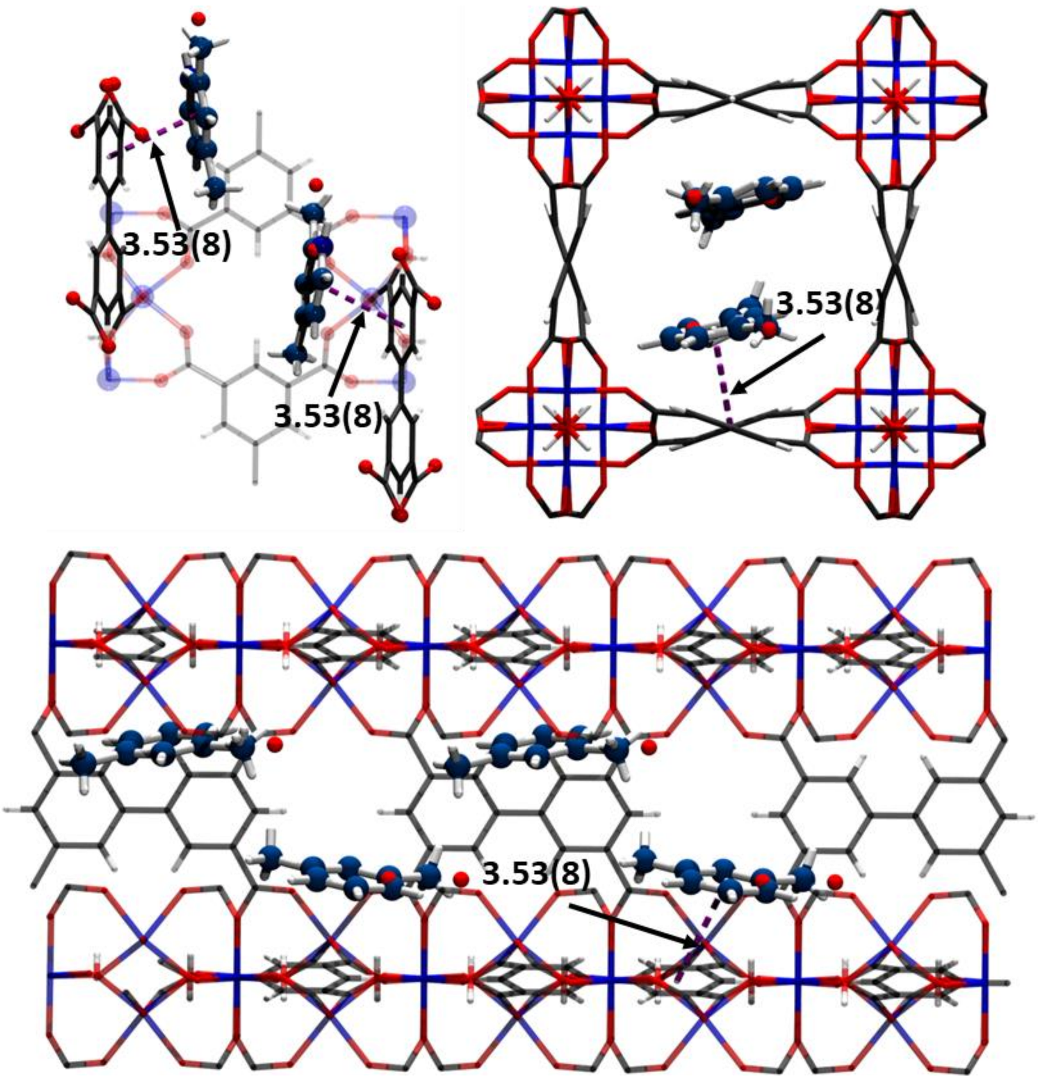

Supplementary Fig. 28. Refined *m*-xylene positions in MFM-300(Al) and three views of the structure of MFM-300(Al)[*m*-xylene]$_{0.50}$. The structure was solved from high-resolution PXRD data. The hydrogen atoms of xylene molecules have been omitted for clarity (aluminium, blue; carbon, grey; oxygen, red; hydrogen, white). The π···π interactions between aromatic rings of xylene molecules and ligands are highlighted in violet dashed lines; the distance is 3.53(8) Å. The occupancy is determined as 0.25.

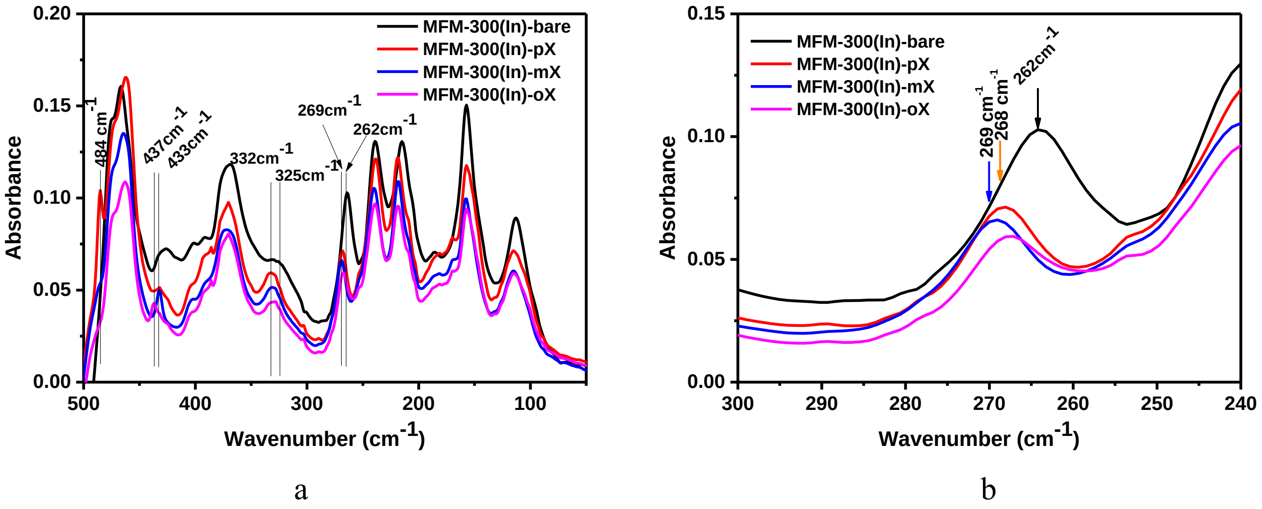

Supplementary Fig. 29. **a,** Comparison of terahertz spectra of bare and xylene-loaded single crystals of MFM-300(In). **b,** detailed terahertz spectra from 300 cm^{-1} to 240 cm^{-1}.

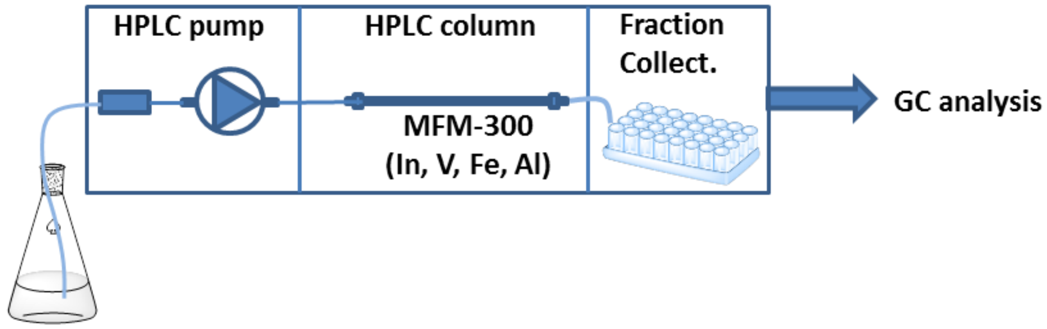

Supplementary Fig. 30. Setup for HPLC and breakthrough experiments.

Supplementary References

1. Hutter, J., Iannuzzi, M., Schiffmann, F. & VandeVondele, J. CP2K: Atomistic simulations of condensed matter systems. *WIREs Comput. Mol. Sci*. **4,** 15-25 (2014).
2. Lippert, G., Hutter, J. & Parrinello, M. A. Hybrid Gaussian and plane wave density functional scheme. *Mol. Phys*. **92,** 477–487 (1997).
3. Vandevondele, J., Krack. M., Mohamed, F., Parrinello, M., Chassaing, T. & Hutter, J. Quickstep: Fast and accurate density functional calculations using a mixed Gaussian and plane waves approach. *Comput. Phys. Commun*. **167,** 103–128 (2005).
4. VandeVondele, J. & Hutter, J. Gaussian basis sets for accurate calculations on molecular systems in gas and condensed phases. *J. Chem. Phys*. **127,** 114105 (2007).
5. Goedecker, S., Teter, M. & Hutter, J. Separable dual-space Gaussian pseudopotentials. *Phys. Rev. B* **54,** 1703-1710 (1996).
6. Perdew, J.P., Burke, K. & Ernzerhof, M. Generalized gradient approximation made simple. *Phys. Rev. Lett.* **77,** 3865-3868 (1996).
7. Grimme, S., Antony, J., Ehrlich, S. & Krieg, H. A consistent and accurate *ab initio* parametrization of density functional dispersion correction (DFT-D) for the 94 elements H-Pu. *J. Chem. Phys.* **132,** 154104 (2010).
8. Krishna, R. Diffusion of binary mixtures in microporous materials: overshoot and roll-up phenomena. *Int. Comm. Heat Mass Transf.* **27,** 893–902 (2000).
9. Vermoortele, F. et al. *P*-xylene-selective metal-organic frameworks: a case of topology-directed selectivity. *J. Am. Chem. Soc.* **133,** 18526–18529 (2011).
10. Moreira, M. A. et al. Reverse shape selectivity in the liquid-phase adsorption of xylene isomers in zirconium terephthalate MOF UiO-66. *Langmuir* **28,** 5715–5723 (2012).
11. El Osta, R. et al. Liquid-phase adsorption and separation of xylene isomers by the flexible porous metal-organic framework MIL-53(Fe). *Chem. Mater.* **24,** 2781–2791 (2012).
12. Alaerts, L. et al. Selective adsorption and separation of xylene isomers and ethylbenzene with the microporous vanadium(IV) terephthalate MIL-47. *Angew. Chem. Int. Ed.* **46,** 4293–4297 (2007).
13. Gonzalez, M. I. et al. Separation of xylene isomers through multiple metal site interactions in metal-organic frameworks. *J. Am. Chem. Soc.* **140,** 3412–3422 (2018).
