# Supplementary material for: Refinement of pore size at sub-angstrom precision in robust metal–organic frameworks for separation of xylenes
Source: Nat Commun. 2020 Aug 27;11:4280. doi: 10.1038/s41467-020-17640-4 (PMC7453017; doi:10.1038/s41467-020-17640-4)
Supplement: Supplementary file 3 — Description of Additional Supplementary Files [file 41467_2020_17640_MOESM3_ESM.docx]

**Legends for Supplementary Movies**

Supplementary Movie 1: Molecular dynamics simulation of *para-*xylene within MFM-300

Supplementary Movie 2: Molecular dynamics simulation of *ortho*-xylene within MFM-300

Supplementary Movie 3: Molecular dynamics simulation of *meta*-xylene within MFM-300
